# A gene-tree test of the traditional taxonomy of American deer: the importance of voucher specimens, geographic data, and dense sampling

**DOI:** 10.3897/zookeys.697.15124

**Published:** 2017-09-14

**Authors:** Eliécer E. Gutiérrez, Kristofer M. Helgen, Molly M. McDonough, Franziska Bauer, Melissa T. R. Hawkins, Luis A. Escobedo-Morales, Bruce D. Patterson, Jesús E. Maldonado

**Affiliations:** 1 PPG Biodiversidade Animal, Centro de Ciências Naturais e Exatas, Av. Roraima n. 1000, Prédio 17, sala 1140-D, Universidade Federal de Santa Maria, Santa Maria, RS 97105-900, Brazil; 2 Departamento de Zoologia, Universidade de Brasília, 70910-900 Brasília, DF, Brazil; 3 Division of Mammals, National Museum of Natural History, Smithsonian Institution, Washington DC, USA; 4 Center for Conservation Genomics, National Zoological Park, Smithsonian Institution, Washington DC, USA; 5 School of Biological Sciences and Environment Institute, University of Adelaide, Adelaide, South Australia 5005, Australia; 6 Museum of Zoology, Senckenberg Natural History Collections, Dresden, Germany; 7 Instituto de Biología, Universidad Nacional Autónoma de México, circuito exterior s/n, Ciudad Universitaria, Coyoacán, CP04510, Mexico City, Mexico; 8 Integrative Research Center, Field Museum of Natural History, Chicago, IL60605, USA; 9 Environmental Science & Policy, George Mason University, 4400 University Dr., Fairfax, VA 22030, USA

**Keywords:** Deer, Cervidae, Neotropics, Americas, Taxonomy, *Odocoileus*, *Mazama*, *Pudu*, *Hippocamelus*, phylogenetics, mDNA, CYTB

## Abstract

The taxonomy of American deer has been established almost entirely on the basis of morphological data and without the use of explicit phylogenetic methods; hence, phylogenetic analyses including data for all of the currently recognized species, even if based on a single gene, might improve current understanding of their taxonomy. We tested the monophyly of the morphology-defined genera and species of New World deer (Odocoileini) with phylogenetic analyses of mitochondrial DNA sequences. This is the first such test conducted using extensive geographic and taxonomic sampling. Our results do not support the monophyly of *Mazama*, *Odocoileus*, *Pudu*, *M. americana*, *M. nemorivaga*, *Od. hemionus*, and *Od. virginianus*. *Mazama* contains species that belong to other genera. We found a novel sister-taxon relationship between “*Mazama*” *pandora* and a clade formed by *Od. hemionus columbianus* and *Od. h. sitkensis*, and transfer *pandora* to *Odocoileus*. The clade formed by *Od. h. columbianus* and *Od. h. sitkensis* may represent a valid species, whereas the remaining subspecies of *Od. hemionus* appear closer to *Od. virginianus*. *Pudu* (*Pudu*) *puda* was not found sister to Pudu (Pudella) mephistophiles. If confirmed, this result will prompt the recognition of the monotypic *Pudella* as a distinct genus. We provide evidence for the existence of an undescribed species now confused with *Mazama americana*, and identify other instances of cryptic, taxonomically unrecognized species-level diversity among populations here regarded as *Mazama temama*, “*Mazama*” *nemorivaga*, and *Hippocamelus antisensis*. Noteworthy records that substantially extend the known distributions of *M. temama* and “*M.*” *gouazoubira* are provided, and we unveil a surprising ambiguity regarding the distribution of “*M.*” *nemorivaga*, as it is described in the literature. The study of deer of the tribe Odocoileini has been hampered by the paucity of information regarding voucher specimens and the provenance of sequences deposited in GenBank. We pinpoint priorities for future systematic research on the tribe Odocoileini.

## Introduction

The tribe Odocoileini (Cervidae: Capreolinae) represents a monophyletic group encompassing all modern deer native to the New World (Americas) with the exception of the Holarctic taxa *Alces alces* (), *Cervus canadensis* (Cervini), and *Rangifer tarandus* (Rangiferini) (Price et al. 2005, Gilbert et al. 2006, Hughes et al. 2006, Agnarsson and May-Collado 2008, Decker et al. 2009, Hassanin et al. 2012, Heckeberg et al. 2016)—see Heckeberg et al. (2016) for current suprageneric taxonomy. Living Odocoileini deer have been traditionally classified in six genera (*Blastocerus*, *Hippocamelus*, *Mazama*, *Odocoileus*, *Ozotoceros*, and *Pudu*) and 16 species (Merino and Rossi 2010, Mattioli 2011; see also Gutiérrez et al. 2015), but alternative taxonomic propositions have suggested that the alpha-level diversity of the tribe might be higher (Molina and Molinari 1999, Molinari 2007, Groves and Grubb 2011). Some authors have also included *Rangifer* as a member of Odocoileini (e.g., Groves and Grubb 2011).

The native distribution of Odocoileini ranges from northern North America (Alaska, Canada) to southern South America (Patagonia), including some islands of the Caribbean Sea and the Atlantic and Pacific oceans. Collectively, members of the tribe occupy a wide variety of habitats, including desert scrub, savannas, swamps, lowland rain forests, humid-montane forests, páramo, and alpine tundra at elevations from sea level to about 4800 meters (Allen 1915, Hershkovitz 1982, Baker 1984, Méndez 1984, Brokx 1984, Medellín et al. 1998, González et al. 2002, Cronin et al. 2006, Meier and Merino 2007, Molinari 2007, Rumiz et al. 2007, Latch et al. 2009, Miranda et al. 2009, Piovezan et al. 2010, Groves and Grubb 2011, Mendes-Oliveira et al. 2011, Barrio 2013, Gutiérrez et al. 2015). By virtue of this wide ecogeographic range, Odocoileini is of great biogeographic interest.

Despite being heavily hunted animals in the Western Hemisphere and also of great public health interest (Bennett and Robinson 2000, Hurtado-Gonzales and Bodmer 2004, Angers et al. 2006, Campbell and VerCauteren 2011, Martinsen et al. 2016, Uehlinger et al. 2016), relatively little progress has been achieved in recent decades with regard to the systematics of Odocoileini deer. To date, only the genera *Mazama* (Allen 1915) and *Pudu* (Hershkovitz 1982) have been subjects of specimen-based revisionary taxonomic work, but these studies did not employ explicit phylogenetic methods. In general, the scientific community has largely followed the taxonomic arrangements recognized by 20^th^ century authorities, predominantly E. R. Hall for North America (Hall 1981) and A. Cabrera for South America (Cabrera 1961). The uncritical acceptance of these taxonomic arrangements for decades is indefensible because the criteria, data, and methods used to construct them are largely unknown, unclear, or even incorrect (see example pointed out by Molinari [2007, p. 31]). Several recent taxonomic studies have demonstrated that the traditional taxonomy of Odocoileini deer needs to be revisited. For instance, morphometric analyses and differences in the frequency of qualitative skeletal traits in *Odocoileus virginianus* of northern South America and North America led Molina and Molinari (1999) to propose that populations from North and South America are not conspecific. These authors also demonstrated a remarkable degree of morphological variability among Venezuelan populations of *Od. virginianus*, whose taxonomy remains disputed (Moscarella et al. 2003, 2007, Molinari 2007). Another example comes from phylogenetic analyses of molecular data demonstrating that the genus *Mazama*, as traditionally understood (Allen 1915, Cabrera 1961), is polyphyletic (Gilbert et al. 2006, Duarte et al. 2008, Hassanin et al. 2012, Escobedo-Morales et al. 2016, Heckeberg et al. 2016). Unfortunately, phylogenetic studies of Odocoileini published to date have been based on limited taxonomic and/or geographic sampling—i.e., lacking taxa or using exemplars for widely distributed and highly variable taxa (e.g., species of *Odocoileus*). Nevertheless, these and other taxonomic studies, some based on karyology (e.g., Jorge and Benirschke 1977, Duarte and Jorge 2003, Cursino et al. 2014), have documented the need to revise the systematics of Odocoileini deer.

Biologically meaningful species-level taxonomies are essential for study design in evolutionary biology, and inadequate species-level classifications, such as uncritically lumping or splitting taxa in absence of appropriate evidence, can detrimentally impact species conservation (George and Mayden 2005, Gutiérrez and Helgen 2013, Heller et al. 2013, Kaiser et al. 2013, Zachos et al. 2013, Voigt et al. 2015, Gippoliti et al. 2017). Accordingly, our long-term goal is to improve all aspects related to the systematics of odocoileines. A first step is to test whether phylogenetic analyses of mtDNA sequence data support the monophyly of recognized genera and species. These analyses have the potential to identify or indicate (1) distant phylogenetic relationships and deep divergences in species or populations currently lumped into a single genus or species, respectively; and (2) close phylogenetic relationships and shallow divergences in species or populations currently split into different genera or species, respectively. Discovering any of these conditions can help target taxa requiring closer attention by taxonomists. Such a test also affords the first assessment of phylogenetic relationships among odocoileines that is simultaneously based on data for all traditionally recognized species, relatively dense geographic sampling within their ranges, and informed by our morphological examination of relevant voucher material in most cases. Nevertheless, because phylogenetic relationships can only be convincingly inferred based on sequence data from multiple, independently inherited loci—e.g., mtDNA, nuclear introns and exons located on different chromosomes—we understand the need to avoid overinterpretations of the gene tree that resulted from our analyses. As interpreted here, our results represent a set of explicit hypotheses that will serve to guide further research.

## Methods

### Sources of material, and taxonomic and geographic sampling

Our analyses were based on 192 sequences of the mitochondrial cytochrome-*b* (*CYTB*) gene. We drew on this marker for three reasons. First, *CYTB* sequences can be obtained relatively easily from degraded DNA that is extracted from museum specimens, which is important for our study since no freshly-preserved samples were available for several targeted species or populations. Second, previous studies have shown that analyses of *CYTB* sequence data can substantially clarify the taxonomic status of mammals whose taxonomy had been predominantly studied based only on morphological and/or karyological data (Duarte et al. 2008, Helgen et al. 2009, Gutiérrez et al. 2010, 2014, 2015, Voss et al. 2013). This coding gene evolves relatively rapidly yet is stable enough to offer insights at suprageneric levels (Agnarsson and May-Collado 2008, Ge et al. 2014), and many studies employing *CYTB* alongside unlinked nuclear sequences have found compatible patterns of variation among them, indicating that *CYTB* can be useful as a first-order estimator of phylogenetic history (Velazco and Patterson 2013, Voss et al. 2014, Upham and Patterson 2015). Third, a large number of *CYTB* sequences are available from GenBank and include most of our focal species. We obtained 171 sequences from GenBank and generated the remaining 21 sequences. All but two (KY928656, KY928667) of the latter sequences were obtained from degraded DNA extracted from museum specimens, from residual soft tissue attached to skeletons, or from maxilloturbinate bones (Wisely et al. 2004) (Table 1). Use of museum specimens allowed us to obtain sequence data for (1) species for which molecular data were previously lacking (i.e., *Mazama chunyi* and *Pudu mephistophiles*; but see Heckeberg et al. 2016), and (2) populations from regions never included in any phylogeographic or phylogenetic study—e.g., from southern Central America and the Andes of Ecuador and Peru for *Odocoileus virginianus*. A study just published by Heckeberg et al. (2016) included *CYTB* data obtained from European museum specimens for *Mazama chunyi* and *Pudu mephistophiles* that we could not access during the development of the present study. We independently generated and analyzed data for these species. We analyzed our sequences employing a more comprehensive geographic sampling for most Odocoileini taxa; hence, we take the opportunity to compare results from both studies and discuss the effect of geographic sampling on the resolution of the gene-trees and its impact on associated taxonomic interpretations. We deposited all sequences that we generated in GenBank, along with the museum catalogue numbers of their respective voucher specimens, tissue numbers, or both (Table 1). The geographic provenance and the names of the institutions that house voucher specimens are provided in the supplementary file 1 (see also Figures 1a, 1b, 1c for abbreviated provenance locality information and GenBank accession numbers of all analyzed sequences).

**Table 1.** **Sequenced specimens.** GB: GenBank accession number. Catalogue#: museum catalogue number. Provenance: geographic origin (name of country, larger administrative entity, and a numeric identifier that corresponds to detailed locality information presented in the Gazetteer; supplementary file 1). DNA: number assigned to DNA extracted. Year: year in which the specimen was collected. M: Sequencing method (I: Illumina; S: Sanger; see Methods).

Species	GB	Catalogue#	Provenance	DNA	Year	M
*B. dichotomus*	KY928652	FMNH 52329	Brazil: São Paulo (3)	EEG 343	1941	I
*M. americana*	KY928653	AMNH 67109	Peru: Cajamarca (10)	EEG 437	1924	I
*M. americana*	KY928654	USNM 443588	Venezuela: Yaracuy (21)	EEG 636	1967	I
*M. chunyi*	KY928655	FMNH 79912	Peru: Puno: Sandia (12)	EEG 297 [MTRH 293]	1951	S
*M. gouazoubira*	KY928656	KU 155307	Guyana: Potaro-Siparuni (8)	EEG 568	1997	I
*M. nemorivaga*	KY928657	AMNH 96171	Brazil: Para (2)	EEG 470	1931	I
*M. nemorivaga*	KY928658	USNM 374916	Venezuela: Bolívar (20)	EEG 628	1966	I
*Od. pandora*	KY928659	KU 93857	Mexico: Campeche (13)	EEG 570	1963	I
*M. rufina*	KY928660 ^1^	FMNH 70563 ^2^	Colombia: Cundinamarca (5)	EEG 326	1952	I
*M. temama*	KY928661	KU 82215	Guatemala: Petén (7)	EEG 572	1960	I
*Od. virginianus*	KY928662	AMNH 62872	Ecuador: Los Ríos (6)	EEG 374	1922	S
*Od. virginianus*	KY928663	AMNH 29453	Nicaragua: Jinotega (16)	EEG 398	1909	S
*Od. hemious*	KY928664	USNM 99455	USA: Arizona (18)	EEG 672	1900	I
*Od. hemious*	KY928665	USNM 249424	USA: Alaska (17)	EEG 666	1930	I
*Od. virginianus*	KY928666	USNM 99351	Mexico: Chihuahua (14)	EEG 039	1899	I
*Od. virginianus* ^3^	KY928667	–	USA: Washington DC (19)	WTD0028	2010	S
*Od. virginianus*	KY928668	FMNH 78421	Peru: Puno (11)	EEG 227	1950	I
*Od. virginianus*	KY928669	KU 149129	Honduras: Cortes (9)	EEG 559	1955	I
*Od. virginianus*	KY928670	KU 93852	Mexico: Yucatán (15)	EEG 562	1963	S
*Oz. bezoarticus*	KY928671	FMNH 28297	Brazil: Mato Grosso (1)	EEG 354	1927	I
*P. mephistophiles*	KY928672	AMNH 181505	Colombia: Cauca (4)	EEG 362	1958	S

^1^ A previous study (Gutiérrez et al. 2015) generated a CYTB sequence (GenBank accession number is KR107038) for this specimen employing Sanger sequencing procedures.

^2^ The museum abbreviation for this specimen has been mistakenly reported as “USNM” (see Supporting information in Gutiérrez et al. 2015). ^3^ Hybrid, cross between *Od. virginianus* and *Od. hemionus* (see Discussion).

**Figure 1a.**
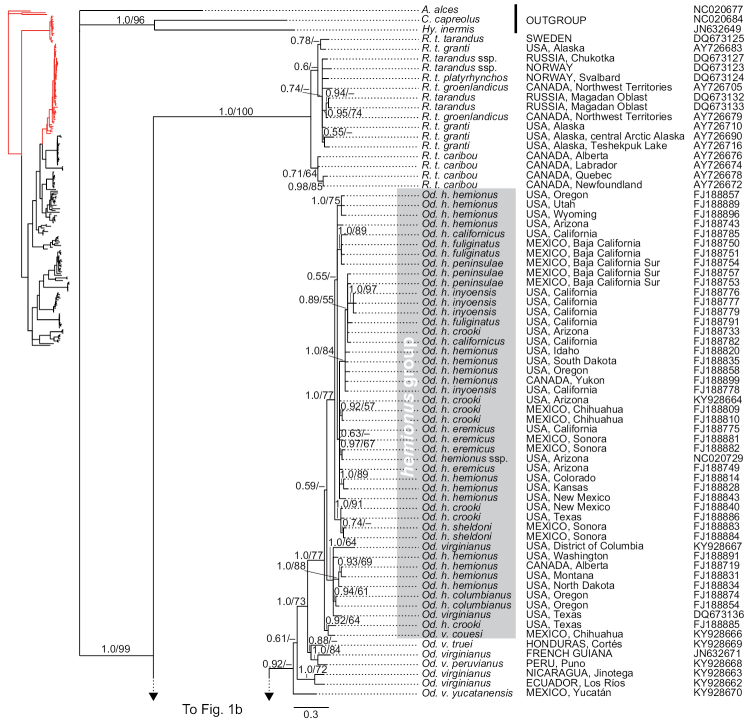
Phylogenetic tree of cytochrome-*b* sequences of Odocoileini. This is a strict consensus topology resulting from the Bayesian inference analysis. Nodal support is indicated at each node, except where the relationship received negligible support. Posterior probabilities (from the Bayesian inference analysis) and bootstrap values (from the maximum-likelihood analysis) are indicated before and after the slashes (“/”) at branches of interest (i.e., nodal support for fairly shallow relationships within intraspecific haplogroups are omitted). The scale represents substitutions per site. For each terminal, country of origin and next-largest administrative unit (state, department, province, etc.) are provided (when reported by the team that generated them; see detailed voucher and locality information in supplementary file 1 for sequences that we generated). GenBank accession numbers are indicated for each terminal.

**Figure 1b.**
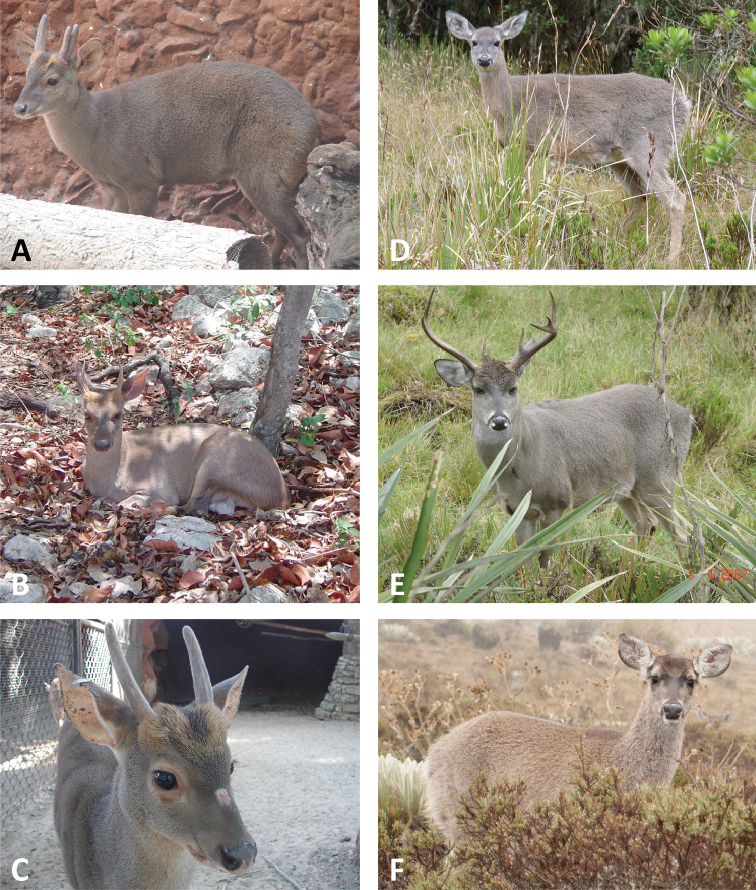
Phylogenetic tree of cytochrome-*b* sequences of Odocoileini (continuation). This is a strict consensus topology resulting from the Bayesian inference analysis. Nodal support is indicated at each node, except where the relationship received negligible support. Posterior probabilities (from the Bayesian inference analysis) and bootstrap values (from the maximum-likelihood analysis) are indicated before and after the slashes (“/”) at branches of interest (i.e., nodal support for fairly shallow relationships within intraspecific haplogroups are omitted). The scale represents substitutions per site. For each terminal, country of origin and next-largest administrative unit (state, department, province, etc.) are provided (when reported by the team that generated them; see detailed voucher and locality information in supplementary file 1 for sequences that we generated). GenBank accession numbers are indicated for each terminal.

**Figure 1c.**
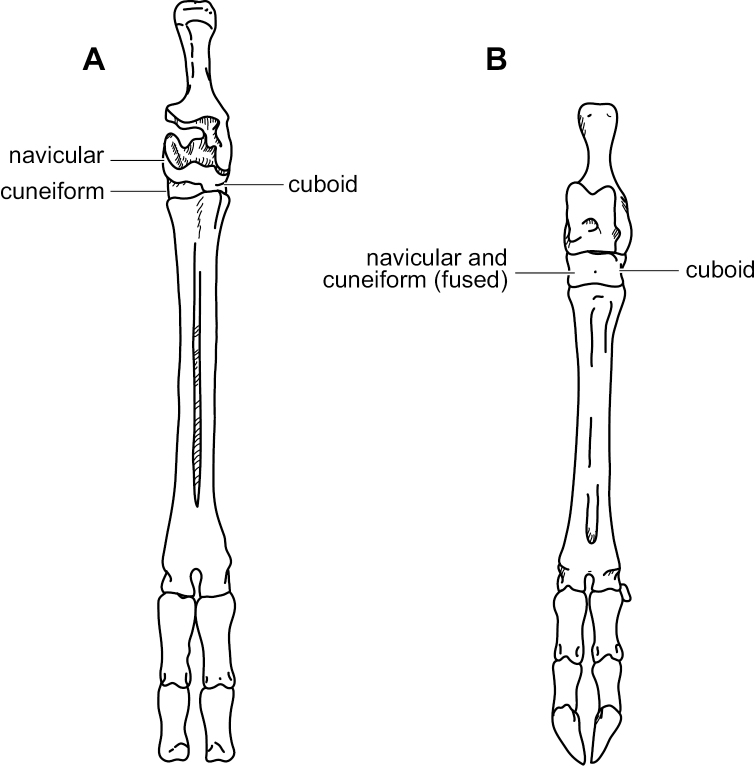
Phylogenetic tree of cytochrome-*b* sequences of Odocoileini (continuation). This is a strict consensus topology resulting from the Bayesian inference analysis. Nodal support is indicated at each node, except where the relationship received negligible support. Posterior probabilities (from the Bayesian inference analysis) and bootstrap values (from the maximum-likelihood analysis) are indicated before and after the slashes (“/”) at branches of interest (i.e., nodal support for fairly shallow relationships within intraspecific haplogroups are omitted). The scale represents substitutions per site. For each terminal, country of origin and next-largest administrative unit (state, department, province, etc.) are provided (when reported by the team that generated them; see detailed voucher and locality information in supplementary file 1 for sequences that we generated). GenBank accession numbers are indicated for each terminal.

### Laboratory methods

We employed both Sanger (following Gutiérrez et al. 2015) and massively parallel (following Hawkins et al. 2016) sequencing technologies to generate part of the analyzed sequences. In order to minimize the risk of contamination with exogenous DNA, all pre-amplification procedures—i.e., DNA extractions, and either settings of conventional PCR reactions or library preparations—based on material obtained from museum specimens were conducted in an isolated facility dedicated exclusively to work with degraded DNA (i.e., where no PCR products have ever been present). We conducted phenol/chloroform DNA extractions following Wisely et al. (2004). Samples were concentrated with Amicon (Millipore, Darmstadt, Germany) filters via centrifugation and stored in siliconized tubes with an additional 20–50 μl of 1 X TE plus 0.5% Tween 20 (Sigma) and stored at -20°C. The DNA of the single freshly preserved tissue sample was extracted in a standard DNA extraction laboratory with a DNeasy Blood and Tissue kit (Qiagen, Valencia, CA, USA) following the manufacturer’s instructions.

We employed various combinations of primers to amplify and to sequence short *CYTB* fragments (supplementary file 2). These reactions were conducted in a six-stage touchdown protocol using a thermal cycler (MJ Research). After an incubation at 95°C for 10 min, the first stage consisted of 2 cycles of the following steps: denaturing at 95°C for 15 seconds, annealing at 60°C for 30 seconds, and extension at 72°C for 1 min. The subsequent stages were identical to the first stage except for lowered annealing temperatures, which were 58°C, 56°C, 54°C, and 52°C for the second, third, fourth, and fifth stages, respectively. The sixth (final) stage consisted of 40 cycles with an annealing temperature of 50°C. All PCR reactions were set in 25 μl volumes containing 0.5 U AmpliTaq Polymerase (Applied Biosystems, Foster City, CA), 1X PCR AmpliTaq Buffer, 0.2 μM each dNTP, 0.4 μM of forward and 0.4 μM of reverse primers, 1.5 μM MgCl2, 10X BSA (New England Biolabs, Ipswich, MA), and 50–250 ng of genomic DNA template. Successful amplifications were purified using ExoSAP (USB Corporation, Cleveland, OH) incubated at 37°C for 15 min followed by 80°C for 15 min. Both strands of each PCR product were cycle sequenced by subjecting them to a second amplification using a total of 10 μL sequencing reaction mixture, including 50–200 ng of PCR product, 10 pM of corresponding forward or reverse primer, 5X Big Dye Buffer (Applied Biosystems), 1/8 reaction of Big Dye version 3 (Applied Biosystems). The following conditions were used for the Dye Terminator Cycle Sequencing: 25 cycles consisting of denaturing at 96°C for 10 s, annealing at 50°C for 10 s and extension at 60°C for 4 min. The final products were cleaned using Sephadex filtration and then both the 3’ and 5’ strands were sequenced on a 50 cm array using the ABI PRISM 3130 Genetic Analyzer (Applied Biosystems). To compile and edit the sequences that were generated via Sanger sequencing, we employed Geneious v.7.1.5. (Biomatters; http://www.geneious.com/).

Some of the analyzed *CYTB* sequences were trimmed from 31 mitochondrial genomes (mitogenomes), 16 obtained from GenBank (generated by Hassanin et al. 2012) and 15 generated by us (following Hawkins et al. 2016). To generate these mitogenomes, we prepared samples for Illumina sequencing using commercially available library preparation kits (Kapa Biosystems Illumina Library Preparation Kit #KK8232, Wilmington, MA, USA). Single indexed TruSeq-style adapters were used (Faircloth and Glenn 2012) employing 50 μl of DNA extract. Minor modifications to the manufacturer’s protocol (see Supplementary Materials Hawkins et al. [2016]) were made, including additional PCR cycles on degraded samples (18 cycles for degraded DNA from museum samples, and 10 for the freshly-preserved DNA sample). The success of library preparation was determined by visualization on an agarose gel. Then, the samples were purified with MagNA magnetic beads (Rohland and Reich 2012) in place of AMPure XP beads to bind DNA and remove the primer and adapter dimer. A ratio of 2.4:1 of MagNA beads to DNA was added to remove adapter dimer. DNA concentration was determined using the Nanodrop v.2.0.

We multiplexed samples in order to decrease the costs associated with library enrichments. Individual samples were multiplexed in equimolar ratios for enrichment based on Nanodrop values in conjunction with the appearance and size distribution from the agarose gel. Each multiplexed pool contained 4–10 uniquely indexed samples for a total concentration of 500 ng concentrated to 3.4 μl volume. The pools also included non-cervid samples from other projects (see Hawkins et al. 2016). We enriched each pool of samples using a probe set that was diluted 1:5, giving each multiplexed pool approximately 100 ng of probes per enrichment. The probes employed corresponded to the same array described by Hawkins et al. (2016). Each pool of libraries was incubated with the RNA probes and buffers as described in the MYcroarray protocol for 48 hours at 65°C. Following incubation, DNA was separated from the probes via magnetic beads and purified with QiaQuick PCR Purification Kits (Qiagen) following MYcroarray’s enrichment protocol (version 1.3.8). Detailed protocols for MYbaits kits have been published online (http://ultraconserved.org/#protocols; http://www.mycroarray.com/pdf/MYbaits-manual.pdf). Post-enrichment pools were amplified for 25 cycles to produce a high enough concentration for gel extraction. QiaQuick Gel Extraction Kits (Qiagen) were used to size select the enriched pools for ~200–500 bp fragments and to remove residual adapter and primer dimer. Quantitative PCR was performed on enriched pools using an Illumina Library Quantification Kit (Kapa Biosystems) with two replicates of 1:1000, 1:2000, and 1:4000 dilutions for each pool. Pools were combined in equimolar ratios based on the number of samples in each pool. The samples were sequenced with paired-end chemistry and with a read length of 143 bp on a single lane of an Illumina HiSeq2500 at the Semel Institute UCLA Neurosciences Genomics Core; reads were demultiplexed at the core facility.

To assemble the mitogenomes, we first merged the forward and reverse paired reads with the program PEAR v0.9.4. (Zhang et al. 2014). Using the default settings of PEAR, we merged forward and reverse reads when they had a 10 bp or greater overlap. All sequences were screened for the presence of adapter sequences, which were removed with cutadapt v.1.4.2 (Martin 2011). We then employed PRINSEQ-lite v.0.20.4 (Schmieder and Edwards 2011) for quality filtering, trimming reads with average quality scores below 20 and exact PCR replicates (more than three identical copies). The filtered reads were then mapped to a reference sequence of the most closely related species using bwa v.7.10 (Li and Durbin 2009). The ‘bwa aln’ and ‘samse’ as well as the ‘bwa mem’ algorithms were tested on the degraded samples, with ‘bwa aln’ conducted as specified in Kircher et al. (2012). The reads corresponding to the freshly preserved tissue sample were mapped using the ‘bwa mem’ algorithm.

### Sequence alignment, matrix properties, and selection of partition scheme and models of nucleotide substitution

We aligned sequences using default options of MAFFT v.7.017 (Katoh and Standley 2013) as implemented in Geneious v.7.1.5. Multiple substitutions in a DNA site (i.e., saturation) compromise historical information from it; therefore, we evaluated whether our *CYTB* matrix suffered from this undesirable condition. Thus, we employed the software DAMBE version 5.3 (Xia 2013) to generate saturation plots based on the GTR-corrected genetic distances. Subsequently, we used the Bayesian Information Criterion (BIC) as implemented in PartitionFinder ver. 1.0.1. (Lanfear et al. 2012) to determine the most suitable partition scheme and best-fit models of nucleotide substitution. This analysis considered models of nucleotide substitution applicable in MrBayes and evaluated five partition schemes.

### Phylogenetic analyses

We conducted phylogenetic analyses using maximum likelihood (ML) and Bayesian inference (BI) as optimality criteria. For all analyses, we employed one sequence of each of the closest related taxa to the Odocoileini—*Alces alces*, *Capreolus capreolus*, and *Hydropotes inermis* (Gilbert et al. 2006, Hassanin et al. 2012)—as outgroup taxa. However, we included *Rangifer* (tribe Rangiferini) as part of the ingroup to test whether it was recovered sister to the clade formed by undisputed Odocoileini (as found in more limited previous studies). Because *Rangifer* has also been treated as a member of Odocoileini by some authors (Groves and Grubb 2011; but see Heckeberg et al. 2016), we take the opportunity to perform the same set of analyses that we are conducting for Odocoileini also for *Rangifer*. For inferring the best topology in the ML analysis, we conducted 50 independent searches in the Genetic Algorithm for Rapid Likelihood Inference (GARLI 2.0) (Zwickl 2006) applying the best-fitting model (see Results) and the default settings. The Bayesian analysis was conducted in MrBayes v. 3. 2 (Ronquist et al. 2012). The search started with a random tree, and the Markov chains were run for 100,000,000 generations; trees were sampled every 1,000 generations. Default values were kept for the ‘‘relburnin’’ and ‘‘burninfrac’’ options in MrBayes (i.e., we used the commands relburnin = yes; burninfrac = 0.25); therefore, the first 25,000,000 generations (25,000 trees) were discarded as burn-in, and posterior probability estimates of all model parameters were based on the remaining (75,000) trees. Convergence and stationarity were assessed in the Bayesian analyses by plotting likelihood values in Tracer 1.5 (Drummond and Rambaut 2007).

To assess nodal support, we used nonparametric bootstrapping (Felsenstein 1985) for the ML analysis, and posterior probabilities for the BI analysis (Ronquist et al. 2012). The ML bootstrap analysis was performed in GARLI 2.0 using 100 pseudoreplicated data matrices, with 10 searches performed on each. Bayesian posterior probabilities were calculated simultaneously with the search for the best Bayesian topology, conducted as described earlier. Throughout the text, we refer to different degrees of nodal support for the ML bootstrap analysis using the following categories: *strong support*, for bootstrap values ≥ 75%; *moderate support*, for bootstrap values > 50% and < 75%; *negligible support* for values ≤ 50%. For the BI analysis, we refer to degrees of nodal support with two categories, *significant* or *strong* in cases in which a node’s posterior probability was ≥ 0.95, and *insignificant* or *negligible* posterior probability values < 0.95.

We assessed the strength of phylogenetic evidence for species boundaries in the *CYTB* tree employing various statistics calculated via the Species Delimitation plugin (Masters et al. 2011) of Geneious v.7.1.5. This plugin allows users to assign terminals of a phylogenetic tree to putative species, which we did using traditional taxonomy of Odocoileini (see Introduction). Based on these designations and the recovered tree, Geneious’ Species Delimitation plugin calculates various statistics relating to the phylogenetic exclusivity of each putative species, the probabilities of such exclusivity having arisen by chance in a random coalescent process, and the degree to which the species can be diagnosed (Masters et al. 2011). The calculated metrics are abbreviated and defined as follows (from Masters et al. 2011): *Intra*, the average pairwise tree distance among members of the focal haplogroup; *Inter*, the average pairwise tree distance between the focal haplogroup and the members of the closest haplogroup; *Intra/Inter*, the ratio of Intra to Inter; *P ID (strict)*, the mean (95% confidence interval) probability of correctly identifying an unknown member of the focal haplogroup using the criterion that it must fall within, but not sister to, the species clade in a tree; *P ID (liberal)*, the mean (95% confidence interval) probability of correctly identifying an unknown member of the putative species using the criterion that it falls within, or sister to, the species clade in a tree; *Av (MRCA-tips)*, the mean distance between the most recent common ancestor of a haplogroup and its members. We computed these statistics twice, once based on the ML tree and another based on the BI tree.

A high degree of sequence divergence is neither necessary nor sufficient for species recognition (Ferguson 2002, Dávalos and Russell 2014); however, as pointed out by Gutiérrez et al. (2010), values of sequence divergence do provide a heuristically useful basis for comparing genetic variation within and among lineages and can help identify taxa in need of closer taxonomic attention. Therefore, we report average uncorrected (p) distance and average Kimura 2-parameter-corrected (K2P) distance within and among haplogroups. Whether justified or not, the latter metric has become widely used in mammals, and therefore we report it to facilitate comparisons with values reported for other groups and by other researchers. Genetic distances were calculated using MEGA version 5.2.1 (Tamura et al. 2011).

## Results

### Alignment properties, partition schemes, and models of nucleotide substitution

The saturation plot demonstrated that the sequence data used in this study do not suffer from saturation; the number of transversions is substantially lower than the number of transitions, even at the highest values of genetic distances (supplementary file 3). The alignment contained 11% missing data. The most suitable partitioning scheme was that in which the three codon positions were analyzed together (i.e., without using subsets). The best-fit model of nucleotide substitution was the generalized time-reversible model with gamma-distributed rate heterogeneity and a proportion of invariant sites (GTR + Г + I).

### Monophyly of traditionally recognized genera

The topologies of the two phylogenetic analyses were similar; we show only the tree resulting from the Bayesian inference analysis (BI) (Figures 1a, 1b, 1c), with nodal support for both the BI and maximum-likelihood (ML) analyses. We comment on the few instances in which results from the two analyses differ. In both analyses, the genera *Blastocerus*, *Hippocamelus*, *Ozotoceros*, and *Rangifer* were recovered as monophyletic with strong support, whereas the genera *Mazama*, *Odocoileus*, and *Pudu* were not (Figures 1a, 1b, 1c; see also column “*Focal haplogroup support*” on Tables 2 and 3). *Mazama* was recovered as polyphyletic, with *Mazama americana* (type species of the genus *Mazama*), *M. bororo*, *M. nana*, *M. pandora*, *M. rufina*, and *M. temama* showing a closer relationship to *Odocoileus* than to the other three species of *Mazama*, namely *M. chunyi*, *M. gouazoubira*, and *M. nemorivaga*, which were recovered elsewhere in the tree. These latter three species showed closer relationships to the genera *Blastocerus*, *Hippocamelus*, *Ozotoceros*, and *Pudu* than to *Odocoileus*. With regard to the genus *Odocoileus*, it was recovered as paraphyletic with respect to *M. pandora* (Figures 1a, 1b), which was recovered sister to a haplogroup containing, almost exclusively, sequences of *Od. hemionus columbianus* and *Od. h. sitkensis* (hereafter referred as the *columbianus* group; Figure 1b). However, the relationship between *M. pandora* and the *columbianus* group received negligible support in both analyses. Lastly, neither analysis supports the monophyly of the genus *Pudu* as currently recognized (Figure 1c). In the BI analysis, our only sequence of *P. mephistophiles* was part of a polytomy that included also a haplogroup formed by *M. nemorivaga* and a clade formed by haplogroups of *M. gouazoubira*, *Blastocerus*, *Hippocamelus*, *Ozotoceros*, and *Pudu puda*. In the ML analysis, this latter multi-genus clade and *P. mephistophiles* were recovered as sister groups with negligible support.

**Table 2.** Summary statistics from the Species Delimitation plugin of Geneious for haplogroups of Rangiferini and Odocoileini deer recovered in the maximum-likelihood tree. Focal haplogroup support: bootstrap values; Intra: The average pairwise tree distance among members of the focal haplogroup; Inter: the average pairwise tree distance between the focal haplogroup and the members of the closest haplogroup; Intra/Inter: the ratio of Intra to Inter; P ID (strict): the mean (95% confidence interval) probability of correctly identifying an unknown member of the focal haplogroup using the criterion that it must fall within, but not sister to, the species clade in a tree; P ID (liberal): the mean (95% confidence interval) probability of correctly identifying an unknown member of the putative species using the criterion that it falls within, or sister to, the species clade in a tree; Av (MRCA-tips): the mean distance between the most recent common ancestor of a haplogroup and its members.

Focal Haplogroup	Closest Haplogroup	Support	Intra	Inter	Intra/Inter	P ID (strict)	P ID (liberal)	Av (MRCA-tips)
*B. dichotomus*	*M. gouazoubira*	100	0.003	0.156	0.02	0.92 (0.80, 1.0)	0.98 (0.87, 1.0)	0.0025
*H. antisensis*	*H. bisulcus*	NA	NA	0.069	NA	NA	0.96 (0.83, 1.0)	NA
*H. bisulcus*	*H. antisensis*	100	0.002	0.069	0.03	0.57 (0.43, 0.72)	0.96 (0.81, 1.0)	0.0011
*americana* group 1	*M. temama*	<50	0.050	0.090	0.56	0.83 (0.77, 0.88)	0.96 (0.93, 0.98)	0.0341
*americana* group 2	*hemionus* group	<50	0.036	0.093	0.39	0.75 (0.65, 0.86)	0.91 (0.85, 0.97)	0.0247
*M. chunyi*	*M. gouazoubira*	NA	NA	0.046	NA	NA	0.96 (0.83, 1.0)	NA
*M. gouazoubira*	*M. chunyi*	61	0.015	0.046	0.32	0.87 (0.80, 0.94)	0.96 (0.92, 1.0)	0.0107
*M. nemorivaga*	*americana* group 2	100	0.069	0.177	0.39	0.78 (0.70, 0.87)	0.93 (0.88, 0.98)	0.0749
*M. pandora*	*columbianus* group	100	0.002	0.111	0.02	0.78 (0.61, 0.96)	1.00 (0.85, 1.0)	0.0013
*M. rufina*	*americana* group 2	93	0.041	0.130	0.32	0.79 (0.69, 0.90)	0.92 (0.86, 0.99)	0.0449
*M. temama*	*americana* group 1	99	0.016	0.090	0.18	0.88 (0.80, 0.97)	0.96 (0.91, 1.0)	0.0270
*hemionus* group	*americana* group 2	<50	0.016	0.093	0.17	0.94 (0.88, 0.99)	0.98 (0.95, 1.0)	0.0246
*columbianus* group	*hemionus* group	100	0.006	0.097	0.06	0.97 (0.92, 1.0)	0.99 (0.97, 1.0)	0.0040
*Oz. bezoarticus*	*M. gouazoubira*	100	0.011	0.138	0.08	0.96 (0.89, 1.0)	0.99 (0.95, 1.0)	0.0111
*P. mephistophiles*	*Oz. bezoarticus*	NA	NA	0.160	NA	NA	0.96 (0.83, 1.0)	NA
*P. puda*	*Oz. bezoarticus*	100	0.004	0.173	0.02	0.97 (0.89, 1.0)	1.00 (0.95, 1.0)	0.0044
*R. tarandus*	*americana* group 2	100	0.010	0.213	0.05	0.98 (0.93, 1.0)	1.00 (0.97, 1.0)	0.0071

### Monophyly of traditionally recognized species

Taxa traditionally regarded as valid species for which we included multiple samples were all recovered as monophyletic with strong support in both analyses (ML, BI), with four exceptions: *Mazama americana*, *M. nemorivaga*, *Odocoileus hemionus*, and *Od. virginianus* (Figures 1a, 1c). Two clades were identified for *M. americana*, and these clades were not sister to each other. One of these clades was formed by haplotypes from Bolivia, Brazil, French Guiana, Paraguay, Peru, and Venezuela, and a strongly supported subclade of *M. bororo* and *M. nana*; hereafter we refer to that clade as the *M. americana* group 1. The monophyly of the *M. americana* group 1 (including as members *M. bororo* and *M. nana*) received negligible and strong support in the ML and BI analyses, respectively. The second clade of *M. americana* included haplotypes from Amazonas, Pará, and southern states of Brazil; hereafter we refer to this clade as the *M. americana* group 2. Monophyly of the *M. americana* group 2 received negligible and moderate support in the ML and BI, respectively. *Mazama americana* group 2 was recovered as sister to a large clade containing *Odocoileus*, *M. pandora*, *M. temama*, and the *M. americana* group 1. In the case of *M. nemorivaga*, all but one sequence were recovered in a fully supported haplogroup that was sister to a single sequence of that species, but this relationship received negligible support (Figure 1c).

Neither of the traditionally recognized species of the genus *Odocoileus* were recovered as monophyletic in any of our analyses. Both analyses recovered most sequences of *Od. hemionus* in a large, strongly supported haplogroup, which also included three sequences from North American *Od. virginianus* (Figure 1a); hereafter we refer to this clade as the *hemionus* group. As mentioned earlier, both analyses also recovered most of the samples attributed to *Od. h. columbianus* and all of the samples attributed to *Od. h. sitkensis* in another fully supported haplogroup (Figure 1b). This haplogroup also included a sequence attributed to *Od. h. hemionus*, though this sample is from Alaska. This haplogroup, as previously mentioned, was found sister to *M. pandora*, albeit with negligible support. Lastly, *Od. virginianus* was not recovered as monophyletic; a few sequences of *Od. virginianus* nested within the *hemionus* group. The remaining sequences of *Od. virginianus* were recovered as closely related to the *hemionus* group, but they did not form supported haplogroups or show clear geographic patterns of relatedness.

### Gene tree-based species delimitation statistics and genetic distances

Species delimitation statistics and genetic distances aided in identifying taxa or haplogroups of taxonomic interest. A low degree of within-haplogroup tree distance suggests that the implicated haplogroup might comprise a single species. The average within-haplogroup tree distances were 0.007 and 0.132 as calculated with the ML and BI trees, respectively. The smallest within-haplogroup tree distances corresponded to *Hippocamelus bisulcus*, *Mazama pandora*, *Blastocerus dichotomus*, and *Pudu puda*, whereas the highest within-haplogroup tree distances corresponded to the *M. americana* group 2, *M. rufina*, *M. americana* group 1, and *M. nemorivaga* (see “Intra” in Tables 2 and 3). Conversely, high tree distances between closely related haplogroups suggest that the haplogroups might not be conspecific. The average between-haplogroup tree distances were 0.115 and 1.512 as calculated with the ML and BI trees, respectively. The smallest between-haplogroup tree distances were those between the two species of *Hippocamelus*, and between *M. chunyi* and *M. gouazoubira*, whereas the highest between-haplogroup tree distances were those between the *columbianus* and *hemionus* groups of the genus *Odocoileus*, and between *M. pandora* with respect to the *columbianus* group (see “Inter” in Tables 2 and 3). Two other metrics, “P ID (strict)” and “P ID (liberal)”, show probabilities of correctly identifying an unknown member of the focal haplogroup using the criteria that it must fall either within or sister to the focal haplogroup, respectively. The lower these probabilities, the less likely that the focal haplogroup represents a valid species. The mean P ID (strict) were 0.856 and 0.849 as calculated with the ML and BI trees, respectively; in both cases only four species had probabilities equal or above 0.95—*Oz. bezoarticus*, *P. puda*, the *columbianus* group, and *R. tarandus* (Tables 2 and 3). The mean values of P ID (liberal) were 0.966 and 0.963 as calculated with the ML and BI trees, respectively; in both analyses all species had probabilities equal or above 0.95, with exception of *M. americana* group 2, *M. rufina*, and *M. nemorivaga* (Tables 2 and 3). Another statistic calculated was the average distance between the most recent common ancestor of a focal haplogroup and the tips of its members, Av (MRCA-tips). The smaller the value of this metric, the more likely members of the focal haplogroup are conspecific. The mean Av (MRCA-tips) were 0.019 and 0.282 as calculated with the ML and BI trees, respectively; in both analyses *H. bisulcus*, *M. pandora*, and *B. dichotomus* showed the smallest Av (MRCA-tips) and *M. rufina* and *M. nemorivaga* the largest (Tables 2 and 3).

Mean uncorrected sequence divergence within species-level haplogroups—provisionally treating the *hemionus* group, the *columbianus* group, the *americana* group 1, and the *americana* group 2 as if each represented an individual species-level haplogroup—ranges from 0.0 to 3.6% (Table 4). However, sequence divergences across the basal split within some species are considerably higher than these average within-group values. In particular, Central American sequences of *Mazama temama* differ from the single available Colombian sample of that species by 5.0%, and the lone sequence of *M. nemorivaga* from the state of Pará, in northern Brazil, differs from all other sequences of that species by 8.3%. Although not a basal split within *M. nemorivaga*, it is noteworthy that *M. nemorivaga* group 1 (from the Guiana Shield) and *M. nemorivaga* group 2 (from Brazil and Peru) differ from one another by 5.9%. Average interspecific divergences within three consistently recovered sister-species pairs (*Hippocamelus antisensis* + *H. bisulcus*, *M. chunyi* + *M. gouazoubira*, and *M. pandora* + *Od. columbianus* group) range from 1.8% to 6.2% (Table 4). The sister-species pair formed by *M. bororo* and *M. nana* was embedded within the diversity of the *M. americana* group 1; the level of divergence between these two species (*bororo* and *nana*) was only 1.3%.

**Table 3.** Summary statistics from the Species Delimitation plugin of Geneious for haplogroups of Rangiferini and Odocoileini deer recovered in the Bayesian tree. Focal haplogroup support: posterior probability values; Intra: The average pairwise tree distance among members of the focal haplogroup; Inter: the average pairwise tree distance between the focal haplogroup and the members of the closest haplogroup; Intra/Inter: the ratio of Intra to Inter; P ID (strict): the mean (95% confidence interval) probability of correctly identifying an unknown member of the focal haplogroup using the criterion that it must fall within, but not sister to, the species clade in a tree; P ID (liberal): the mean (95% confidence interval) probability of correctly identifying an unknown member of the putative species using the criterion that it falls within, or sister to, the species clade in a tree; Av (MRCA-tips): the mean distance between the most recent common ancestor of a haplogroup and its members.

**Focal Haplogroup**	**Closest Haplogroup**	**Support**	**Intra**	**Inter**	**Intra/Inter**	**P ID (strict)**	**P ID (liberal)**	**Av (MRCA-tips)**
*B. dichotomus*	*M. gouazoubira*	1.00	0.065	2.014	0.03	0.91 (0.79, 1.0)	0.98 (0.87, 1.0)	0.0352
*H. antisensis*	*H. bisulcus*	NA	NA	0.906	NA	NA	0.96 (0.83, 1.0)	NA
*H. bisulcus*	*H. antisensis*	1.00	0.047	0.906	0.05	0.56 (0.41, 0.71)	0.95 (0.80, 1.0)	0.0236
*americana* group 1	*M. temama*	0.95	0.688	1.248	0.55	0.83 (0.78, 0.88)	0.96 (0.93, 0.98)	0.4722
*americana* group 2	*M. temama*	0.89	0.509	1.334	0.38	0.76 (0.65, 0.86)	0.91 (0.85, 0.98)	0.3445
*M. chunyi*	*M. gouazoubira*	NA	NA	0.639	NA	NA	0.96 (0.83, 1.0)	NA
*M. gouazoubira*	*M. chunyi*	0.95	0.250	0.639	0.39	0.85 (0.78, 0.91)	0.95 (0.91, 1.00)	0.1888
*M. nemorivaga*	*P. mephistophiles*	0.92	0.939	2.198	0.43	0.77 (0.68, 0.85)	0.93 (0.87, 0.98)	0.9906
*M. pandora*	*columbianus* group	1.00	0.050	1.437	0.03	0.77 (0.59, 0.94)	0.99 (0.84, 1.0)	0.0305
*M. rufina*	*americana* group 2	1.00	0.585	1.794	0.33	0.79 (0.68, 0.89)	0.92 (0.86, 0.98)	0.6342
*M. temama*	*americana* group 1	1.00	0.239	1.248	0.19	0.88 (0.79, 0.96)	0.96 (0.91, 1.0)	0.3774
*hemionus* group	*americana* group 2	0.92	0.270	1.391	0.19	0.93 (0.87, 0.98)	0.98 (0.95, 1.0)	0.4257
*columbianus* group	*hemionus* group	1.00	0.117	1.416	0.08	0.96 (0.91, 1.0)	0.99 (0.96, 1.0)	0.0617
*Oz. bezoarticus*	*M. gouazoubira*	1.00	0.190	1.885	NA	0.95 (0.88, 1.0)	0.98 (0.94, 1.0)	0.1755
*P. mephistophiles*	*americana* group 2	NA	NA	1.921	0.00	NA	0.96 (0.83, 1.0)	NA
*P. puda*	*Oz. bezoarticus*	1.00	0.084	2.063	0.04	0.96 (0.88, 1.0)	1.00 (0.94, 1.0)	0.0454
*R. tarandus*	*americana* group 2	1.00	0.179	2.658	0.07	0.97 (0.92, 1.0)	0.99 (0.96, 1.0)	0.1416

**Table 4.** Matrix of genetic distances (percent sequence divergence) within and among recovered haplogroups of Rangiferini deer. Average uncorrected (p) distances among conspecific sequences are arrayed along the diagonal, interspecific p distances are below the diagonal, and Kimura two-parameter (K2P) distances are above the diagonal. No genetic distances were calculated within species for which we only had a single sequence available; however, we duplicated each of these sequences to allow for calculations of interspecific p-distances. The following names apply to haplogroups (as recovered in our phylogenetic analyses) rather than to species: *Mazama americana* 1, *M. americana* 2, *hemionus* group, and the *columbianus* group.

	1	2	3	4	5	6	7	8	9	10	11	12	13	14	15	16	17
**1. *Blastocerus dichotomus***	**0.3**	4.4	7.6	9.8	10.3	7.1	7.2	6.9	10.9	8.0	10.0	8.4	9.0	8.2	5.2	14.6	9.5
**2. *Hippocamelus antisensis***	4.2	—	3.0	8.8	9.3	2.5	2.6	5.8	9.9	7.3	8.8	7.4	8.0	8.3	4.2	12.5	6.6
**3. *Hippocamelus bisulcus***	7.1	2.9	**0.8**	8.3	9.9	3.8	5.8	5.9	11.4	5.6	8.3	10.1	9.5	7.8	7.4	10.2	5.6
**4. *Mazama americana* 1**	9.1	8.2	7.7	**2.8**	3.7	7.2	9.1	8.9	4.3	4.8	3.3	6.6	4.8	10.3	7.8	10.1	6.9
**5. *Mazama americana* 2**	9.5	8.5	9.1	3.8	**3.2**	7.2	8.8	9.0	4.3	5.2	3.9	6.7	5.8	10.6	8.3	11.1	8.6
**6. *Mazama chunyi***	6.7	2.5	3.7	6.7	6.8	–	1.8	6.3	7.0	4.7	6.0	7.5	7.1	9.3	7.0	11.7	7.6
**7. *Mazama gouazoubira***	6.8	2.6	5.4	8.4	8.1	1.8	**0.5**	7.1	9.0	6.5	8.0	7.7	9.1	11.4	7.1	13.4	9.6
**8. *Mazama nemorivaga***	6.5	5.5	5.6	8.2	8.3	5.9	6.6	**3.6**	9.6	5.3	8.0	7.8	9.4	8.1	6.6	12.2	9.2
**9. *Mazama pandora***	10.0	9.0	10.2	4.1	4.1	6.6	8.3	8.8	**0.0**	6.3	5.2	7.5	6.2	13.3	8.8	12.9	9.7
**10. *Mazama rufina***	7.5	6.8	5.3	4.6	5.0	4.5	6.1	5.1	5.9	**1.5**	3.7	6.7	6.4	8.3	6.3	8.4	6.7
**11. *Mazama temama***	9.3	8.2	7.8	3.2	3.8	5.7	7.5	7.5	5.0	3.6	**0.7**	5.6	4.6	8.7	8.0	9.9	6.8
**12. *columbianus* group**	7.9	7.0	9.2	6.2	6.3	7.0	7.2	7.2	6.9	6.3	5.4	**2.2**	6.6	12.3	6.3	11.9	9.1
**13. *hemionus* group**	8.4	7.5	8.7	4.5	5.5	6.7	8.4	8.7	5.8	6.0	4.4	6.2	**0.2**	11.3	7.0	11.0	7.9
**14. *Ozotoceros bezoarticus***	7.7	7.7	7.2	9.5	9.7	8.5	10.3	7.5	11.8	7.7	8.0	11.1	10.3	**0.7**	9.2	15.2	7.8
**15. *Pudu mephistophiles***	5.1	4.1	7.0	7.3	7.7	6.6	6.7	6.2	8.2	5.9	7.5	6.0	6.7	8.5	—	10.4	5.8
**16. *Pudu puda***	13.1	11.3	9.4	9.4	10.2	10.7	12.0	11.1	11.7	7.9	9.2	10.8	10.1	13.5	9.7	**0.4**	9.8
**17. *Rangifer tarandus***	8.7	6.1	5.3	6.5	7.9	7.1	8.7	8.4	8.9	6.2	6.4	8.3	7.4	7.3	5.5	9.1	**0.8**

## Discussion

### Polyphyly and phylogenetics of the genus *Mazama*

Based on data from all nine currently recognized species of *Mazama* (Gutiérrez et al. 2015), we confirm the findings by previous authors (Gilbert et al. 2006, Duarte et al. 2008, Hassanin et al. 2012, Escobedo-Morales et al. 2016, Heckeberg et al. 2016) that the genus, as traditionally understood (Allen 1915, Cabrera 1961), is polyphyletic. In the only comprehensive revisionary work published for *Mazama*, Allen (1915) stated that the main characteristics that distinguish the genus *Mazama* from other deer genera are: short, unbranching (spike-like) antlers in males (but note that males of the genus *Pudu* also possesses spike-like antlers); small, slightly expanded bullae in comparison with those of *Odocoileus* and *Blastocerus*; flat and usually nearly straight upper borders of the orbits; slight over-hang of the frontals over the postorbital fossa; overall small size and the red coloration of most of its species; Allen also acknowledged the existence of a group of *Mazama* with brown coloration (Allen 1915). Clearly, our results and those from previous studies, one of them based on multi-locus data, demonstrate that this morphological characterization of *Mazama* does not diagnose a natural group (Gilbert et al. 2006, Escobedo-Morales et al. 2016). Logically, either some of these morphological characteristics resulted from convergent evolution, or they represent plesiomorphies inherited from an ancestor shared by many of these deer. Ancient hybridization, incomplete lineage sorting, or both, often explain lack of monophyly in recently originated clades when limited sequence data are analyzed (particularly mitochondrial DNA data); however, species traditionally classified into the genus *Mazama* are so widely distributed throughout the recovered tree that it seems unlikely that these phenomena explain the observed, rampant polyphyly.

The tribe Odocoileini is divided into two major clades for which subtribe-level names have recently been proposed (Heckeberg et al. 2016). The subtribe Odocoileina contains taxa from temperate, subtropical, and tropical regions of the Americas, whereas the subtribe Blastocerina contains taxa exclusively from subtropical and tropical regions of South America (see Figures 1a, 1b, 1c). In our analyses, both subtribes were recovered with poor nodal support, but their monophyly has been supported by previous studies (e.g., Gilbert et al. 2006, Hassanin et al. 2012). *Mazama*, as traditionally understood, is represented by species in both subtribes. In our analyses, the Odocoileina included all of the species of *Mazama* with red (reddish) pelage, i.e., *M. americana*, *M. bororo*, *M. nana*, *M. rufina*, *M. temama*; one *Mazama* species with brown (brownish/grayish) pelage coloration, *M. pandora*; and the genus *Odocoileus*. The remaining three species of *Mazama* with brown (i.e., brownish or grayish) pelage coloration (i.e., *M. chunyi*, *M. gouazoubira*, *M. nemorivaga*) were recovered in Blastocerina, which also includes the genera *Blastocerus*, *Hippocamelus*, *Ozotoceros*, and *Pudu*. These findings confirm, with more comprehensive sampling, those from two recent mtDNA-based studies (Escobedo-Morales et al. 2016, Heckeberg et al. 2016). Results from these studies clearly call into question the validity and usefulness of the terms “red clade”, “red brocket species group”, “gray clade”, “gray brocket species group”, “brown group”, all of which have been previously applied to groups (e.g., by Allen 1915, Duarte et al. 2008, Escobedo-Morales et al. 2016) whose respective monophyly has never been supported. These terms based on pelage coloration are highly misleading. For example, the term “gray clade” erroneously implies that all of the species now allocated within the subtribe Blastocerina possess predominantly gray pelage coloration, but almost half of the species in this subtribe lack such coloration (*Blastocerus dichotomus*, *Mazama chunyi*, *Ozotoceros bezoarticus*, *Pudu mephistophiles*; Hershkovitz 1959, 1982, Jackson 1987, Rumiz et al. 2007, Miranda et al. 2009), and, more importantly, species of “*Mazama*” with gray pelage were not recovered as a monophyletic group in either our analyses or those of previous studies (Gilbert et al. 2006, Duarte et al. 2008, Escobedo-Morales et al. 2016).

### Phylogenetic relationships and taxonomy of species traditionally classified as *Mazama*

Our results have implications for the alpha-level taxonomy of *Mazama*. Phylogenetic analyses based on *CYTB* data by Duarte et al. (2008) recovered *M. americana* in two distinct haplogroups, one of which also included terminal branches that they identified as *M. bororo* and *M. nana*. In that study, however, these haplogroups formed part of a polytomy together with *Odocoileus* and a sequence of “*Mazama* sp.” Subsequently, based on partial sequences of both the *CYTB* gene and the mitochondrial control-region (D-LOOP), Abril et al. (2010) recovered two monophyletic haplogroups within *M. americana*. Despite the lack of resolution in the results obtained by Duarte et al. (2008), Abril et al. (2010) assumed the monophyly of *M. americana* by the composition of their ingroup (i.e., not including other odocoileines), and, therefore, the topology they obtained could not evaluate whether *M. americana* represents a single species. However, more recent studies employing *CYTB* sequence data from multiple species of Odocoileini have shown *M. americana* to be polyphyletic (Escobedo-Morales et al. 2016, Heckeberg et al. 2016). Based on more comprehensive sampling, our results confirm the polyphyly of *M. americana* (as currently understood) and provide novel insights regarding the possible taxonomic identity and geographic distribution of at least two species currently lumped within *M. americana*. Before discussing this topic, we clarify that comparisons of the *CYTB* sequences generated by Abril et al. (2010) with respect to those analyzed by us indicated that the two haplogroups obtained by the former group of researchers match our *M. americana* groups 1 and 2. Because the name *M. americana* is based on the type locality of Cayenne, French Guiana (see Allen 1915), our *M. americana* group 1, which included a sequence (accession number NC020719; Figure 1b) from Barrage de Petite-Saut (Alexandre Hassanin *in litt.*), northern French Guiana, located at only ca. 80 km E Cayenne, likely corresponds to *M. americana* *sensu stricto*. Further work is necessary to determine whether *M. americana* group 1 truly corresponds to *M. americana* *sensu stricto*. If confirmed, then the sequence data herein analyzed, and that produced by Abril et al. (2010), would document the presence of *M. americana* *sensu stricto* in French Guiana, Bolivia, Brazil (states of Acre, Pará, Paraná, Rondônia, and São Paulo), Paraguay (department of Alto Paraná), Peru (Region of Cajamarca), and Venezuela (state of Yaracuy). The provenance localities of other analyzed samples of *M. americana* group 1 are unknown (see *Caveats*). Further taxonomic work is also necessary to confirm that *M. americana* group 2 is not conspecific with *M. americana* *sensu stricto* and, if so, assign to it a species name. Our analysis documents this lineage (provisionally referred to as “*M. americana* group 2” or “*M. americana* 2”) in the states of Amazonas and Pará in northern Brazil. In addition, a sequence that matches our *M. americana* group 2 was generated by Abril et al. (2010) from a karyotyped individual born in captivity (in “Criadouro Santarém”) in the Brazilian state of Pará, but of unknown geographic origin. Previous research focused on Brazilian populations of *M. americana* *sensu lato* has shown the existence of at least six distinct karyotypes in different regions of that country, and inter-cytotype crosses in captivity demonstrated reproductive isolation among the most geographically-distant cytotypes (Cursino et al. 2014). The results from our phylogenetic analyses are congruent with these karyological and reproductive observations, and confirm that more than a single species is currently lumped under *M. americana* *sensu lato*. To date, the only study that has examined the morphological variation of *Mazama americana* *sensu lato* in a large portion of its distribution is the unpublished master thesis of Dr. Rogério V. Rossi (Rossi 2000). Based on morphometric analyses of Brazilian samples, Rossi found that specimens from littoral areas of southeastern Brazil (from Santa Catarina to São Paulo states) are slightly differentiated from those obtained from populations to the interior of that country. Whether a correspondence exists between these two morphologically distinguished groups and the clades identified in the present study remains to be addressed.

Reconciling current phylogenetic information for *Mazama bororo* and *M. nana* with their taxonomic status as valid species presents a conundrum. The existence of two species of small brockets in southern South America has been noted in the scientific literature since the first half of the 19^th^ century (Lesson 1842, Goeldi 1893, Lydekker 1898, 1915, Miranda-Ribeiro 1919). These deer are currently referred to as *M. bororo*, known from remnants of Atlantic Forest in southeastern São Paulo and eastern Paraná and Santa Catarina, Brazil (Duarte and Jorge 2003, Vogliotti and Duarte 2009, Duarte et al. 2016), and as *M. nana*, known from Atlantic Forest habitat in southern São Paulo, Paraná, Santa Catarina, and northern Río Grande do Sul, Brazil (Rossi 2000, Vivo et al. 2011, Duarte et al. 2012). Records of *M. nana* also exist from the Alto Paraná and Itapúa departments of Paraguay (Gamarra de Fox and Martin 1996) and the Misiones province of Argentina (Di Bitetti et al. 2008). No agreement about their taxonomic status was reached until recently, when they were recognized as valid species on the basis of chromosomal differences between them and with *M. americana* *sensu lato* (Duarte and Jorge 2003, Abril and Duarte 2008). Reported karyotypes for these species include the following diploid and fundamental numbers (2n/FN): *Mazama bororo*: 32–34/46 (Duarte and Jorge 2003); *M. nana*: 36/56, 37/59, 38/60 (Duarte and Jorge 2003), 36–39/58 (Abril and Duarte 2008); *M americana* group 1: 50/54; *M americana* group 2: 42/49, 43/48, 49/56, 51/56 (Duarte and Jorge 2003). Additional karyotypes reported for *M. americana* *sensu lato* lacking *CYTB* sequences are available—and hence not assigned to group 1 or 2—include the following 2n/FNs: 42/46, 43/46, 44/46, 44/48, 45/48, 50/54, 52/56, 53/56 (Abril et al. 2010). These data and a study that involved crosses in captivity to assess hybrids’ fertility have demonstrated that: (1) *Mazama bororo* is not a hybrid between *M. nana* and *M. americana*, and is unable to produce fertile hybrids with either of these species (Duarte and Jorge 2003); and (2) *M. americana* groups 1 and 2 are reproductively isolated (Cursino et al. 2014). Based on these findings, phylogenetic analyses based on a relatively fast-evolving gene would be expected to recover *M. bororo*, *M. nana*, and *M. americana* as independent lineages; however, the former two species were recovered nested within *M. americana* group 1. For species in this complex, future systematic efforts should concentrate in three areas. First, to investigate the phylogeographic structure of populations in the *M. americana* group 1, which implicitly requires assessing the phylogenetic position of *M. bororo* and *M. nana*, based on sequence data from multiple unlinked loci, including nuclear DNA segments with faster mutation rates than the *CYTB* gene to resolve finer-scale relationships. This approach would concomitantly enable assessment of the potential role of hybridization, incomplete lineage sorting, or both as causal explanations for the topology obtained in our analyses (see above). Second, the specific mechanisms responsible for the remarkable karyological variation observed in this group need further investigation, as do their implications for speciation. Although important advances have been made unveiling the chromosomal variation in this group (e.g., Duarte and Jorge 1996, 2003, Abril and Duarte 2008, Cursino et al. 2014), much remains to be done, including investigating the possible role of B chromosomes—which are able to create even intra-individual karyological variation (Abril and Duarte 2008, Abril et al. 2010)—on speciation (if any). The mechanisms that have been postulated to explain the chromosomal variability of *Mazama americana* *sensu lato* need to be revisited because *M. americana* *sensu lato* is not monophyletic, as previously (and implicitly) assumed (by Abril et al. 2010, Cursino et al. 2014). Third, a morphological assessment of differences among natural groups (identifiable by molecular and karyological criteria) should be conducted in search of diagnostic traits. Preliminary analyses of linear measurements taken on craniodental and external traits allow unambiguous discrimination between *M. americana* *sensu lato* and *M. nana*, but not between the former and *M. bororo* (Rossi 2000). This is likely an artifact created by the fact that such comparisons were conducted assuming that populations of *M. americana* *sensu lato* comprised a single species, inflating its apparent variability. Similar comparisons between *M. bororo* and *M. nana* permitted unambiguous discrimination between both of these species (Rossi 2000; but see Duarte et al. 2008).

**Figure 2.**
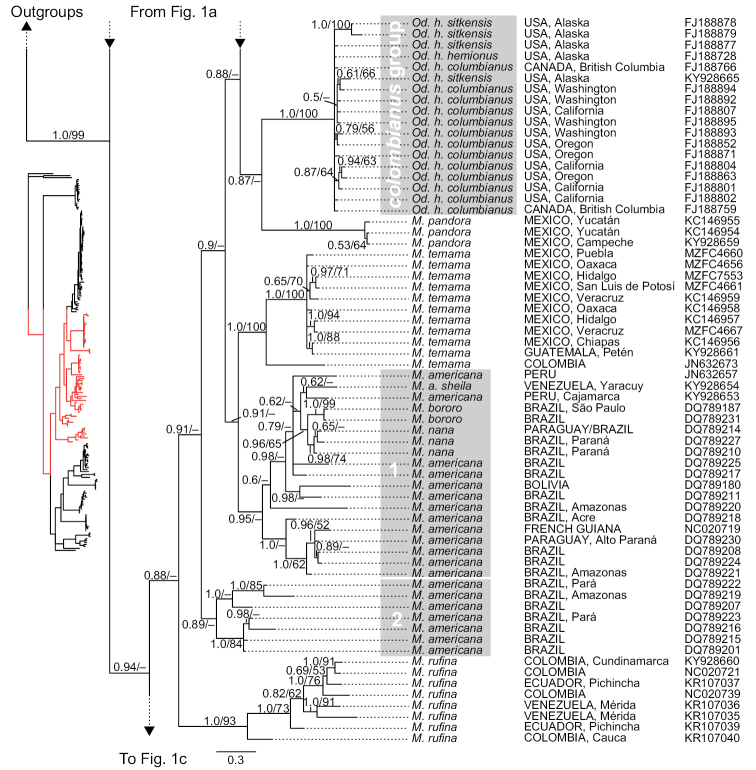
Overall morphological appearance of “*M.*” *pandora* (panels **A–C**) and that of the genus *Odocoileus* (panels **D–F**). Notice the grayish pelage and divergent antlers larger than in other species currently classified in *Mazama*. “*M.*” *pandora*, panels **A** and **C** individuals kept in captivity at the Parque Zoológico del Bicentenario Animaya, Mérida, Yucatán, Mexico (photographs by Luis A. Escobedo-Morales)—provenance unknown; panel **B** individual kept in captivity in Tekax, Yucatán, Mexico (photograph by Rosa María González Marín)—provenance unknown. *Odocoileus virginianus* (see proposals by Molina and Molinari 1999 and Molinari 2007); panels **D** and **E** Monteredondo, Parque Nacional Chingaza, ca. 47 km (by road) E Bogota, Cundinamarca, Colombia (photographs by Aideé Vargas-Espinoza and Irene Aconcha, respectively); panel **F** Laguna de Mucubají, Parque Nacional Sierra Nevada, Mérida, Venezuela (photograph by Rodrigo Díaz Lupanow).

Our results offer novel phylogenetic information with respect to *Mazama pandora*, a species endemic to the Península de Yucatán. A recent study based on mtDNA (Escobedo-Morales et al. 2016) recovered *M. pandora* as a monophyletic haplogroup sister to *Odocoileus virginianus*, the only species of *Odocoileus* analyzed in that study. Another study reanalyzed these and additional data and found *M. pandora* sister to a clade composed by a handful of sequences of *Od. virginianus* and *Od. hemionus* of unspecified geographic origin; the two species of *Odocoileus* were found intermixed with each other within a poorly supported monophyletic group (Heckeberg et al. 2016). Our more comprehensive sampling identified a novel sister-taxon relationship between *M. pandora* and the *columbianus* group—the latter is a clade formed by most *Odocoileus h. columbianus* samples and all samples of *Od. h. sitkensis*, and a sample of *Od. h. hemionus*, whose inclusion in this clade might be a consequence of hybridization. Given the traditional assignment of *pandora* to the genus *Mazama* (Allen 1915, Medellín et al. 1998), its nested position within *Odocoileus* was unexpected. However, the overall morphological appearance of *M. pandora* somewhat resembles that of the genus *Odocoileus* (Figure 2); the species has grayish pelage, and divergent antlers larger than other species classified in *Mazama*. It is expected that future work will unveil morphological synapomorphies between species of *Odocoileus* and *pandora*. The sister relationship between *pandora* and the *columbianus* group also suggests that the biogeographic history of these deer is complex, but this topic requires robust phylogenetic inference, enabling ancestral area reconstructions and proper molecular dating. However, discussing the nomenclatural implications of the close relationship between *pandora* and the genus *Odocoileus* is necessary, especially after Escobedo-Morales et al. (2016) advocated allocating species of *Odocoileus* into the genus *Mazama*. Such an action, which has been contemplated by a few modern authors (Haltenorth 1963, Grubb 2000, Groves and Grubb 2011), would increase congruence between available phylogenetic information and the taxonomic nomenclature of Odocoileini but diminish efficiency in communication of scientific information. Allocating species currently treated as *Odocoileus* within *Mazama* would unnecessarily (see below) disrupt the association between the name *Odocoileus* and at least two—and perhaps more (Molina and Molinari 1999, Molinari 2007)—species epithets and the names of numerous subspecies (between 48 and 71) (Baker 1984, Brokx 1984, Méndez 1984, Smith 1991). This action would pose difficulties for retrieval of data and bibliography from repositories, such as GenBank and the Global Biodiversity Information Facility, and search engines, such as Google Scholar and the Web of Science, respectively. This is not a trivial matter because, given the importance of *Odocoileus* in aspects raging from public health to landscape ecology, massive amounts of data are associated with the name *Odocoileus*, whose North American members are among the most studied ungulates worldwide. A more suitable solution to the current incongruence between the phylogenetic information available and the nomenclature of these deer would be to restrict the use of the name *Mazama* to the clade containing *M. temama* and the *Mazama americana* group 1; to allocate *M. pandora* to the genus *Odocoileus*, and to recognize *M. rufina* and the *M. americana* group 2 as belonging to two separate genera, other than *Mazama*. Disrupting association between the genus and species epithets for “*Mazama*” *pandora*, “*Mazama*” *rufina* and taxa within the *M. americana* group 2 is both unavoidable—because of the polyphyletic nature of *Mazama* (as currently understood)—and less problematic for scientific communication because these species are far less studied than those of *Odocoileus*. This solution would reconcile the available phylogenetic information with the taxonomy of the group while minimizing nomenclatural instability. Similar considerations and actions have been recently employed to preserve binomial stability in various mammalian groups, including opossums (Giarla et al. 2010, Voss et al. 2014, Díaz-Nieto and Voss 2016, Pavan and Voss 2016), rodents (Teta et al. 2016), and primates (Garbino 2015, Gutiérrez and Marinho-Filnho 2017). A third alternative would be to retain *pandora* in *Mazama* until data from independently inherited loci become available. However, no analytical evidence, of any sort, supports a close relationship between *pandora* and *M. americana*, the type species of the genus. Although analyses of data from a single gene offer incomplete bases for taxonomic revisions, they represent an improvement when the traditional taxonomy in question is based on no evidence whatsoever. In those cases, dogmatically preserving the traditional taxonomy would essentially translate into imposing beliefs while ignoring data. The transferral of *pandora* to an already-described genus, *Odocoileus*, seems a sensible and justifiable provisional action, considering not only the phylogenetic evidence here presented but also resemblance in external morphology between *pandora* and species of *Odocoileus* (Figure 2). By contrast, allocating presumed clades currently regarded as *Mazama* sensu lato into different genera would involve either the description of new genera or the recognition of available generic names which are currently treated as junior synonymies, without sufficient consideration of morphological traits that might support such actions. These nomenclatural improvements should be carried out once a robust multi-locus phylogeny becomes available and should be coupled with morphological diagnoses of the genera to be proposed.

Besides confirming the monophyly of *Mazama temama* (Escobedo-Morales et al. 2016), we provide evidence that this species occurs in South America, or that populations in Colombia perhaps represent a currently unrecognized species. Previously, *M. temama* had been regarded as a Central American endemic, ranging from southeastern Mexico to Panama (Allen 1915). However, some authors speculated that the species could also range into northern Colombia, but provided no evidence or explanation (Bello-Gutiérrez et al. 2010). In our analyses, a sequence (GenBank accession number JN632673) from Parque Nacional Chingaza, near Bogotá, Cundinamarca, Colombia (Manuel Ruiz-García *in litt*.), previously assigned to *Odocoileus virginianus* (Hassanin et al. 2012), was recovered as sister to a haplogroup containing sequences of *M. temama* (Figure 1b). Because this latter haplogroup comprised sequences obtained from samples that were correctly identified via examination of voucher specimens (see Escobedo-Morales et al. 2016), we herein re-identify this Colombian sample as *M. temama*. Our finding of the species in Colombia is congruent with unpublished morphometric data obtained by EEG, KMH, and JEM. In their recent study, Escobedo-Morales et al. (2016) retained the identity of sequence JN632673 as *Od. virginianus* (a procedure also followed by Heckeberg et al. 2016) but noted that it could have resulted from misidentification, contamination, or hybridization with other species of *Mazama*, or that it might represent an unnamed species. Our results cannot reject that this sequence belongs to an currently unrecognized species because the sequence divergence existing between sequence JN632673 (from Colombia) and the Central American haplogroup of *M. temama* is (ca. 5.0%) substantially higher than divergences known between sister species pairs of Odocoileini deer (all below 3%; see Results). Hence, our assignment of sequence JN632673 to *M. temama* should be regarded as provisional; further work should explore the possibility that two species might be currently lumped within *M. temama*.

Three species traditionally regarded as members of the genus *Mazama* were recovered within Blastocerina, the subtribe endemic to South America. One of them, *M. chunyi*, has only been incorporated twice in phylogenetic assessments (herein and in the just-published study by Heckeberg et al. 2016), and in each case based on a single *CYTB* sequence (obtained from different specimens). *Mazama chunyi* was found sister to *M. gouazoubira*, which was recovered in a monophyletic haplogroup (with strong and moderate support in the BI and ML analyses, respectively). Thus, pending confirmation via analyses of additional molecular data, it is likely that *M. chunyi* and *M. gouazoubira* represent a sister-species pair: one member is restricted to montane habitats of the Bolivian and Peruvian Andes (*M. chunyi*) and the other is widely distributed in lowland habitats of South America (*M. gouazoubira*). If this result is corroborated, then both species should be assigned to a genus other than *Mazama* (which is based on *Mazama americana* and likely applies to *Mazama americana* group 1, see above). Even if further analyses do not confirm their sister-taxon relationship, both species need to be transferred to a genus other than *Mazama* because they share a most recent common ancestor with members of the subtribe Blastocerina, not with the type species of *Mazama*, which belongs to the subtribe Odocoileina. We note that the genus-group name *Nanelaphus* Fitzinger, 1873, with type species *N. namby* Fitzinger (= *M. gouazoubira*), may be available for this clade (Lydekker 1898, Allen 1915).

We recovered two principal reciprocally monophyletic haplogroups within *Mazama nemorivaga*: one (*M. nemorivaga* 1) formed exclusively by samples from the northern portion of the species’ range—i.e., from the Venezuelan state of Bolivar, the Guyanean region of Potaro-Siparuni, an unknown locality from French Guiana, and the Brazilian state of Rondônia—and the other (*M. nemorivaga* 2) formed by samples from two unknown localities (one from Brazil and another from Peru) and from the Brazilian states of Pará, Paraná, and Rondônia. The monophyly of these haplogroups received either moderate or strong support. *Mazama nemorivaga* was recovered in our analyses as an isolated lineage divergent from other South American lineages of *Mazama*, including the *M. gouazoubira*-*M. chunyi* clade, with which it has been taxonomically associated for most of its past taxonomic history (e.g., Miranda-Ribeiro 1919, Cabrera 1961; but see Allen 1915, Rossi 2000). Further research is needed to confirm its relationships and distinctness, but our results suggest it may require genus-level recognition within the Blastocerina. We note that the generic-level name *Passalites* Gloger, 1841, with type species *Cervus nemorivagus* Cuvier, 1817 (= *M. nemorivaga*), is available for this clade (Palmer 1904).

We found evidence that suggests that habitat association in *Mazama gouazoubira* and *M. nemorivaga* might have impacted their phylogeographic structure in contrasting ways. Despite the wide distribution of *M. gouazoubira*, which apparently ranges from Colombia (see below) to Argentina, we found shallow phylogeographic relationships among analyzed populations of this species (Figures 1a, 1b, 1c). This pattern might be explained by the tolerance of this species to a wide range of environmental conditions, as suggested by its occurrence across dry, wet, forested and open habitat types (Black and Vogliotti 2008, Black-Décima et al. 2010, Duarte et al. 2012). Wide environmental tolerance might have enabled historical connectivity among populations and gene flow. Conversely, in *M. nemorivaga*, a species that seems to be predominantly associated with tropical and subtropical broadleaf moist forest habitats (as described by Olson et al. 2001; Rossi and Duarte 2016), we found substantially deeper phylogeographic pattering. This pattern might be a consequence of past expansion and contractions of wet forest habitats isolating populations. Such expansions and contractions of forest habitats are thought to have triggered vicariance events that shaped the phylogeographic structure observed in species closely associated to either wet forest- or dry forest habitat types (Gutiérrez et al. 2014).

Our analyses also yielded new insights regarding the distribution of “*Mazama*” *gouazoubira*. Given that a Colombian sample of “*M.*” *gouazoubira* (GenBank accession number JN632658 [curated version number NC_020720]; Hassanin et al. 2012), obtained from an live individual from northern Bolívar department (Manuel Ruiz-Garcia, *in litt*.), was recovered nested within a haplogroup containing all other samples of that species, our results demonstrate that the northern limit of the species’ distribution is not the southern margin of the Amazon basin, as recently argued (Black and Vogliotti 2008, Black-Décima et al. 2010, Duarte et al. 2012). The Colombian sample extends the distribution of *M. gouazoubira* at least ca. 1000 km to the north of literature records of the species in northwestern Bolivia (Black and Vogliotti 2008, Black-Décima et al. 2010, Duarte et al. 2012)—this distance is a rough estimate as we were not able to obtain detailed locality information for this sample (see Hassanin et al. 2012).

We take the opportunity to comment on ambiguities that have prevailed in the literature with regard to the distribution of *Mazama nemorivaga*. Important discrepancies exist among published distribution maps for this species. For example, Duarte et al. (2012) depicted a distributional range for the species that includes the Amazonian region and the Guianas, the eastern slopes of the Ecuadorian and Peruvian Andes, the southern half of the Andean cordilleras of Colombia, the Sierra de Santa Marta and lowlands in northern Colombia, and the Lago de Maracaibo basin and the Península de Paraguaná in northwestern Venezuela. However, Rossi and Duarte (2016) omitted the Colombian Andes from their range map for this species, but included the entire Venezuelan mainland with exception of the Andean cordilleras, the Península de Paraguaná, and the northern half of the La Guajira department of Colombia. These differences seem to have resulted from attempts to combine records of *M. nemorivaga* from Amazonia and the Guianas with alleged records of that species from other regions. A modern revisionary work evaluating the taxonomy of brockets in northern South America is indispensable to achieve reliable knowledge on the distribution of *M. nemorivaga* and determine which of the populations in northwestern South America, if any, correspond to *M. nemorivaga*, whose type locality is Cayenne, French Guiana (Allen 1915).

### Monophyly and phylogenetics of the genus *Odocoileus*

Our results do not support the monophyly of the genus *Odocoileus* as traditionally understood because the node shared by all samples of *Odocoileus* received negligible support in both analyses and, more importantly, because *Mazama pandora* was found embedded within *Odocoileus* (as previously discussed). Because of the apparent recent origin of *Odocoileus*, it is likely that recovering the genus and its species as monophyletic groups would require examination of DNA segments with higher mutations rates than that of the *CYTB* gene. In fact, we conducted preliminary analyses (not shown) of sequence data from the mitochondrial control region (D-loop) and *CYTB* generated for a previous study on the phylogeography of *Od. hemionus* (Latch et al. 2009) and found that, when analyzed alone, the *CYTB* data failed to provide an adequately supported topology. By contrast, D-loop sequences analyzed in combination with the *CYTB* data yielded a more structured tree and with better nodal support (similar to that shown in figure 2 of Latch 2009).

### Phylogenetic relationships and taxonomy of species of *Odocoileus*

Our results do not support the monophyly of either of the species traditionally recognized within the genus *Odocoileus*, i.e., *Od. virginianus* and *Od. hemionus*. Two explanations are likely. First, as mentioned above, the substitution rate of *CYTB* appears too low to allow adequate resolution of relationships as recent as these. In other words, incomplete lineage sorting might be responsible for the observed lack of monophyly in these taxa. Second, the observed lack of monophyly in these species is a partial consequence of hybridization between them, a phenomenon that has been widely documented (Carr et al. 1986, Stubblefield et al. 1986, Cronin et al. 1988, Key and Boe 1992, Cathey et al. 1998, Hornbeck and Mahoney 2000, Bradley et al. 2003). Hybridization between *Od. hemionus* and *Od. virginianus*, or among their respective subspecies (e.g., Hopken et al. 2015), seems to occur not only along contact zones of their native ranges, but also in areas to which they have been translocated for commercial purposes. For instance, a free-ranging deer in natural areas within Washington DC (the National Zoo, Smithsonian Institution), with external characteristics matching *Od. virginianus*, had a CYTB haplotype (KY928667) that places it within the *hemionus* group in our gene tree. This is a sign of hybridization between both species in the state of Virginia, where individuals of *Od. hemionus* were translocated decades ago (Linzey 1998). Hybridization can also explain other instances in which nominal taxa were not recovered in monophyletic groups. For example, although most samples of black-tailed deer (*Od. hemionus columbianus* and *Od. h. sitkensis*) form a clade, two sequences attributed to *Od. h. columbianus* from Alaska were not recovered within this clade. These two sequences were recovered within the *hemionus* group which can be attributed to hybridization between *Od. h. columbianus* and other subspecies of *Od. hemionus* (Latch et al. 2009, 2011 and references therein). Similarly, hybridization may also explain why a sequence attributed to *O. h. hemionus* from Alaska was recovered within the *columbianus* group.

The traditional classification of species of *Odocoileus* is incongruent with the phylogenetic information currently available for them. Our results suggest (1) that the *columbianus* and *sitkensis* lineages, currently treated as subspecies of *Od. hemionus*, form a clade that is more closely related to *Od. pandora* than to *Od. hemionus*; and that (2) *Od. hemionus* appears more closely related to *Od. virginianus* (even to *Od. virginianus* from South America!) than to its putative subspecies *columbianus* or *sitkensis*. In agreement with this possibility, the level of uncorrected genetic divergence, calculated with *CYTB* sequence data, between the *hemionus* and the *columbianus* groups (6.2%) greatly exceeds mean levels of divergences within species (and species-like lineages) of Odocoileini and Rangiferini (all below 3.6%, Table 4). Surprisingly in view of their importance to North American hunters, no phylogenetic study using nuclear sequence data from mule deer, white-tailed deer, and black-tailed deer have been conducted to date. If further analyses based on sequence data obtained from independently inherited loci confirm the topology obtained from mtDNA, then reconciling taxonomy with phylogenetics would require elevating *columbianus* and *sitkensis* to species rank (see Future Directions). However, such further analyses based on multiple loci are likely to produce an alternative topology, for example by recovering all lineages of mule deer, white-tailed deer, and black-tailed deer as a monophyletic group and with *pandora* sister to it. Under this plausible scenario, and for the sake of binomial stability, which has important implications for scientific communication (see discussion on this topic by Gutiérrez and Marinho-Filho 2017), we transfer *pandora* to the genus *Odocoileus*, in congruence with the close relationship and overall similarity it shares with other members of *Odocoileus*. Regardless of which of these alternative topologies will be favored by additional analyses, dense geographic sampling is necessary to produce a suitable taxonomic classification with respect to lineages currently treated as members of *Od. hemionus* and *Od. virginianus*. This is particularly important due to the tremendous morphological variation documented among (even geographically close) populations of Neotropical white-tailed deer and the possibility that they might not be conspecific (as proposed by Molina and Molinari 1999, and Molinari 2007).

### Monophyly and phylogenetic relationships of the remaining Blastocerina

According to the traditional taxonomy of Odocoileini deer, the recently described subtribe Blastocerina contains four species-poor genera, *Hippocamelus* and *Pudu* containing two species each, and the monotypic *Blastocerus* and *Ozotoceros*. Our analyses supported the monophyly of *Hippocamelus* and *H. bisulcus*. In addition, none of our tree- or genetic-distance metrics suggests the existence of additional unrecognized species within this genus. The single analyzed sequence of *H. antisensis* did not nest within the haplogroup of any other species. Nevertheless, our sampling for this genus was poor; additional studies might reveal higher diversity within the two traditionally recognized species of *Hippocamelus*. In fact, the recent study by Heckeberg et al. (2016) analyzed the same sequences that we analyzed and two additional sequences of *Hippocamelus antisensis* (of unknown geographic precedence). These authors recovered these additional sequences (hereafter referred to as *H. antisensis* lineage 2) as sister to *Ozotoceros*. A third sequence of that species analyzed by these authors (which we analyzed; hereafter referred to as *H. antisensis* lineage 1) was recovered as sister to *H. bisulcus*. Therefore, their results challenge the monophyly of both the genus *Hippocamelus* and *H. antisensis* (Heckeberg et al. 2016), and suggest that an unrecognized species related to *Ozotoceros* might exist among populations currently assigned to *H. antisensis*. Nevertheless, ancient hybridization and incomplete lineage sorting remain as alternative causal explanations for these results; these possibilities need to be tested with data from unlinked loci.

Our results support the monophyly of both *Blastocerus* and *Ozotoceros*, and none of our tree- or genetic-distance metrics suggest the possible existence of currently unrecognized species within sampled populations currently referred to as *Blastocerus dichotomus* or *Ozotoceros bezoarticus*. These results agree with results from previous studies (González et al. 1998, 2002, Márquez et al. 2006, Duarte et al. 2008). Both of our phylogenetic analyses (BI, ML) recovered *Blastocerus* sister to a clade containing “*Mazama*” *gouazoubira*, “*Mazama*” *chunyi*, and the genus *Hippocamelus* (*H. bisulcus* + *H. antisensis* lineage 1); however, this relationship received insignificant support in the BI analysis and modest support in the ML analysis. That phylogenetic position for *Blastocerus* agrees with that recovered by Heckeberg et al. (2016) from *CYTB* data, but disagrees with the topology obtained by Hassanin et al. (2012) from complete mitochondrial genomes, who recovered *Blastocerus* sister to “*Mazama*” *nemorivaga*. Duarte et al. (2008) found *Blastocerus* sister to a clade formed by *H. bisulcus* and “*Mazama*” *gouazoubira*. A likely explanation for this difference is that these authors used different optimality criteria than the ones that we used. The tree presented by Duarte et al. (2008) seems to have been produced by a neighbor-joining analysis (a phenetic technique) (showing bootstrap values from that analysis and from a Maximum-Parsimony analysis), whereas our analyses were based on Bayesian and Maximum-Likelihood optimality criteria. Duarte et al. (2008) also mentioned that an unreported Bayesian inference analysis they conducted yielded a similar topology to those of their other two analyses. Differences in the taxon sampling used by Duarte et al. (2008) and Hassanin et al. (2012) with respect to our taxon sampling might also help explain the differences between their topologies and ours. Similar factors could also explain disagreement between our results and those from previous studies with regard to the phylogenetic position of *Ozotoceros*. Albeit with negligible support, our analyses recovered *Ozotoceros* as sister to a clade formed by *Blastocerus*, *Hippocamelus*, “*Mazama*” *gouazoubira*, and “*Mazama*” *chunyi*. Both Duarte et al. (2008) and Heckeberg et al. (2016) found *Ozotoceros* sister to a sequence representing *Hippocamelus antisensis* lineage 2, whereas Hassanin et al. (2012) recovered *Ozotoceros* sister to a clade formed by “*Mazama*” *gouazoubira* and *Hippocamelus antisensis* lineage 1.

**Figure 3.**
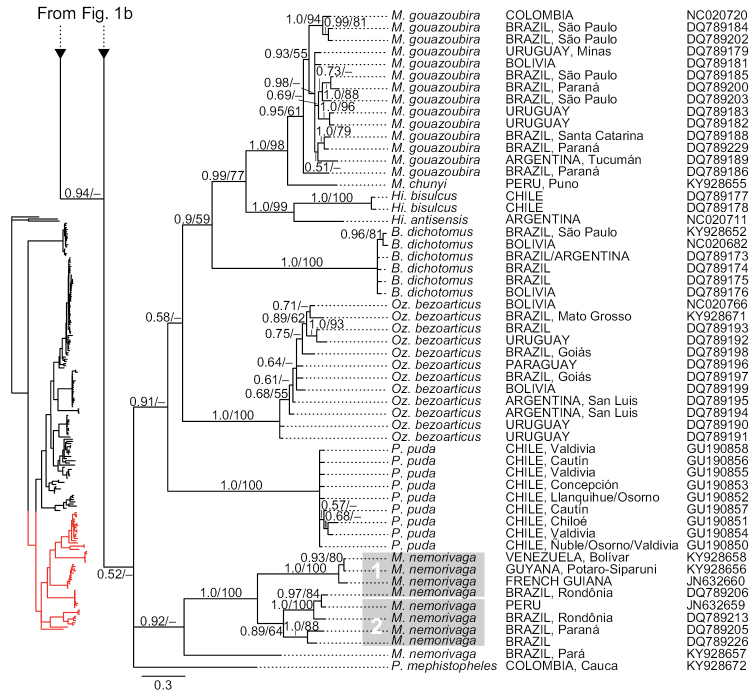
Hind foot bones of *Mazama rufina* (**A**) and *Pudu puda* (**B**) *sensu* Hershkovitz (1982). According to Hershkovitz (1982; see also Brooke 1874, 1878), the union of the cuboideonavicular and external and middle cuneiform tarsal bones into a single bone in *Pudu* is the only osteological characteristic shared by *P. puda* and *P. mephistophiles* that consistently separates them from all other living deer, with exception of the genera *Elaphodus* and *Muntiacus*.

A case deserving close attention concerns the monophyly (or lack thereof) of the genus *Pudu*. According to the traditional taxonomy, *Pudu* contains two species, *P. (Pudu) puda* and *P. (Pudella) mephistophiles* (Hershkovitz 1982). The former occurs in Argentina and Chile, at elevations from sea level up to 1700 meters, whereas the latter occurs in the Andes of Colombia, Ecuador, and Peru at elevations between 1700 and 4000 meters (Hershkovitz 1982, Cronin et al. 2006, Meier et al. 2007, Escamilo et al. 2010, Jiménez 2010). Our results do not support the monophyly of the genus as traditionally recognized. *Pudu puda*, which is the type species of the genus, was recovered sister to a clade including “*Mazama*” *gouazoubira*, “*Mazama*” *chunyi*, *Hippocamelus* (*H. bisulcus* + *H. antisensis* lineage 1), *Blastocerus*, and *Ozotoceros*—this position was recovered in the best tree resulting from the ML analysis and in the consensus tree resulting from the BI analyses, but in both cases with negligible support. This large putative clade (including all the taxa just mentioned) was recovered sister to *P. mephistophiles* in the ML analysis, but with negligible nodal support. The BI analysis recovered *P. mephistophiles* in a polytomy at the base of the subtribe Blastocerina. This polytomy contained two additional branches, one leading to “*Mazama*” *nemorivaga* and another containing all other members of Blastocerina. The recent study by Heckeberg et al. (2016) analyzed multiple partial *CYTB* sequences of *P. mephistophiles*; these authors conducted various analyses, but recovered the species in various positions, including: *P. mephistophiles* as sister to all other Blastocerina (as in our ML analysis); as sister to Odocoileini and Rangiferini; and in an unresolved position with other Odocoileini clades and Rangiferini. However, Heckeberg et al. (2016) also analyzed a sequence labeled as *P. mephistophiles* (by Hassanin et al. 2012), overlooking the observation already made by Gutiérrez et al. (2015), who demonstrated that this sequence actually corresponds to “*Mazama*” *rufina*. Despite the ambiguity regarding the position of *P. mephistophiles*, *P. puda* was consistently recovered in our analyses and in those by Heckeberg et al. (2016) as being more closely related to Blastocerina other than *P. mephistophiles*. This fact suggests the possibility that the genus *Pudu*, as traditionally defined, is not monophyletic. If confirmed by future studies, the monotypic *Pudella* (Thomas 1913), which is currently treated as a subgenus of *Pudu*, would warrant genus rank. According to Hershkovitz (Hershkovitz 1982; see also Brooke 1874, 1878), the union of the cuboideonavicular and external and middle cuneiform tarsal bones into a single bone (Figure 3) is the only osteological characteristic shared by *P. puda* and *P. mephistophiles* that consistently separates them from all other living deer, except from the distantly related Asiatic genera *Elaphodus* and *Muntiacus* (Gilbert et al. 2006, Hassanin et al. 2012). Could this anatomical similarity between *P. puda* and *P. mephistophiles* be the result of evolutionary convergence rather than a trait inherited from a recent common ancestor shared between these two species? Convergence could also explain other similarities between these species, like their small sizes and spike-like antlers, among others (see Hershkovitz 1982). Evolutionary convergence in morphological appearance has misguided supraspecific classifications of deer before, most spectacularly in the case of the genus “*Mazama*” *sensu lato* (as traditionally understood) (see findings of molecular studies based on data from either mDNA, nDNA, or both: Gilbert et al. 2006, Duarte et al. 2008, Hassanin et al. 2012, Escobedo-Morales et al. 2016, Heckeberg et al. 2016, the present study). Regardless of these issues concerning supraspecific classification, our results and those by Heckeberg et al. (2016), support the species-level monophyly of both *P. puda* and *P. mephistophiles*. Both of our phylogenetic analyses recovered *P. puda* in a single strongly supported haplogroup, and none of our analyses recovered the single analyzed sequence of *P. mephistophiles* embedded within another species’ haplogroups. None of our tree- or genetic-distance metrics suggest the existence of species-level diversity currently unrecognized among their populations.

### A word on the genus *Rangifer*

Because we employed dense taxonomic and geographic sampling for Odocoileini deer, we sought to test if our approach confirmed the monophyly of this tribe and therefore included *Rangifer* as part of our ingroup. *Rangifer*, which is currently placed within the subtribe Rangiferini (Heckeberg et al. 2016), has been recovered sister to the Odocoileini in previous studies that were based on limited sampling for both Odocoileini and Rangiferini (Gilbert et al. 2006, Hassanin et al. 2012). We were also able to test, for the first time, the monophyly of *Rangifer* with dense sampling of both *Rangifer* and various Odocoileini. Our results were not controversial, as both of our phylogenetic analyses provided strong support to the monophyly of the genus *Rangifer* and it was found sister to a clade formed by all Odocoileini—this Odocoileini clade was recovered in both analyses, albeit with negligible support in both cases.

### Caveats

Three main caveats affect the present study and, more generally, have hampered progress towards a suitable taxonomy for Odocoileini deer. First, the scarcity of freshly preserved tissue samples for Neotropical deer has restricted many studies to Sanger sequencing technologies and mitochondrial DNA, and in most cases only partial sequences of one or two genes are used. At present, *CYTB* is the only gene sampled broadly enough to support the geographic and taxonomic scope of the present study. Our new *CYTB* sequences filled some geographic and taxonomic gaps pre-existing on GenBank, but not all of them, and particularly for widely distributed taxa (e.g., *Odocoileus virginianus* and *Mazama americana*), data are still missing from large and biogeographically interesting portions of their ranges.

Secondly, the use of sequence data from a single locus is an obvious limitation. Because the mode of inheritance of mitochondrial DNA is matrilineal, our use of *CYTB* sequences allows inference only of matrilineal relationships among sampled populations, which might be contradicted when sequence data from additional loci become available. Nevertheless, because female philopatry is rampant in mammals, matrilineal relationships are useful to identify priority regions and taxa in phylogenetic comparison. Moreover, previous studies based on *CYTB* sequence data have regularly improved the classification of tropical mammalian groups (e.g., Patterson and Velazco 2008, Solari et al. 2009, Gutiérrez et al. 2010, 2015, Voss et al. 2013, Moratelli et al. 2016, 2017, Molinari et al. 2017, the present study) whose decades-old classifications had been based on assessments of morphological similarity. Many of these classifications, including that of Odocoileini deer, were proposed at times predating the implementation of phylogenetic, or even statistical, analyses in taxonomic research.

Third, many of the sequences available from GenBank are not associated with voucher specimens, lack geographic data, or both. This is likely due to the fact that many colleagues that generated these data are not taxonomists—but ecologists, wildlife managers, conservation biologists, and researchers working on public health issues—and they did not need to report such data for their particular research goals. Unfortunately, in many instances, it has not been reported whether voucher specimens are available and, if so, basic information associated with these specimens (e.g., institution in which they are housed, catalogue numbers, criteria used to assign taxonomic identifications) have not been provided. Similarly, geographic provenances of samples used to generate sequence data are rarely reported and, when reported, often limited to names of country and large administrative entities (e.g., state, department, etc.). Moreover, some Neotropical members of the tribe Odocoileini are rare, subject to intense pressure by humans (e.g., due to hunting and habitat loss), or both, which has hindered, in some countries, obtaining permits to collect specimens for research. To circumvent this difficulty, researchers have sometimes resorted to using samples obtained from animals kept in captivity. Often, zoos do not maintain detailed records of the provenance of animals they keep. The ambiguities resulting from all the aforementioned factors compromise the use of such samples (and derived sequences) from certain types of analyses (e.g., ancestral area reconstructions); even when they can be used, these issues often limit the interpretations that could otherwise be made. Examples of the latter type of problem are some of the sequences that we analyzed and that represent new and noteworthy distributional records—e.g., the apparent first record of *Mazama temama* for South America and Colombia; the apparent first record of “*Mazama*” *nemorivaga* for northwestern South America and Colombia—unfortunately, no detailed information about their provenance were published by the research teams that generated these sequences (see discussion above).

### Future directions

Our results suggest that future systematic studies on Odocoileini deer should prioritize assessments of the taxonomic status of populations historically assigned to widely distributed taxa—e.g., species of *Odocoileus* and *Mazama americana*. *Odocoileus virginianus* shows great morphological variability. Regional patterns of this high morphological variability have led authors to propose that multiple species exist among populations traditionally referred to *Od. virginianus* (Molina and Molinari 1999, Molinari 2007). A study based on phylogenetic information and adequate sampling from North, Central, and South America has yet to be conducted to evaluate these proposals. Similarly, efforts based on mtDNA sequences (including the present report) and karyology have advanced our understanding of the variability of *M. americana* and documented the existence of an undescribed species among populations traditionally referred to this taxon. This species needs to be described, a process that necessarily requires both testing our hypothesis that *M. americana* group 1 likely corresponds to *M. americana* *sensu stricto* and solving the current incongruence between phylogenetics and the taxonomy of *M. bororo* and *M. nana*. Other cases in which available phylogenetic information identified the likely existence of undescribed species are those of *Hippocamelus antisensis*, whose populations have been recovered in two lineages that are not sister to each other (Heckeberg et al. 2016), and South American populations provisionally assigned to “*Mazama*” *temama* (the present study). A single sequence of “*Mazama*” *temama* is known from this region, but it is from an unknown locality in Colombia. This sequence is highly divergent from a clade formed by Central American populations of “*Mazama*” *temama*. Future fieldwork in northwestern South America and study of specimens housed at museums, particularly those in Colombia and Venezuela, might provide additional samples of this likely undescribed taxon.

Clearly, substantial species-level taxonomic work is yet to be done. As the scientific community advances tackling the many taxonomic issues of cervid species, researchers should keep in mind that, despite the conservation status of some of these deer and the implicit difficulty to obtaining collecting permits for research, especially in the Neotropics, new species and subspecies should only be described when preserved museum specimens are available to document new names (see Ceríaco et al. 2016, Gutiérrez and Pine 2017, Dubois 2017 and references therein, Pine and Gutiérrez [in press]; contra Donegan 2008, Marshall and Evenhuis 2015). In addition, and also to avoid obstructing scientific progress, upcoming studies should provide sufficient information regarding voucher specimen availability and detailed information regarding the provenance of samples from which they have obtained data; unfortunately, this is not customary.

The current supraspecific taxonomy of Odocoileini deer does not closely align with the information currently available regarding their phylogenetic relationships (Gilbert et al. 2006, Duarte et al. 2008, Hassanin et al. 2012, Escobedo-Morales et al. 2016, Heckeberg et al. 2016, the present study). Further phylogenetic analyses and morphology-based revisionary work is required. The use of massively-parallel sequencing technologies and the unprecedented potential to generate large amounts of DNA data from museum specimens offers the most promising approach to solve this incongruence; however, museum work should also be conducted to enable proper characterization and diagnoses of generic names to be assigned to clades. Efforts to generate a more robust phylogeny will also provide a basis for biogeographic studies on Odocoileini deer. Such studies will be of great interest for understanding aspects of the Great American Biotic Interchange and other major events in the deep history of the American continents. Results presented in this study suggest that some long-lived notions about areas of origin and number and direction of dispersal events of deer are erroneous, but correcting them will require meaningful estimates of times since divergences and ancestral area reconstructions.

## Supplementary materials

Supplementary material 1 Gazetteer Link: https://doi.org/10.3897/zookeys.697.15124.suppl1 Authors: Eliécer E. Gutiérrez, Kristofer M. Helgen, Molly M. McDonough, Franziska Bauer, Melissa T. R. Hawkins, Luis A. Escobedo-Morales, Bruce D. Patterson, Jesús E. Maldonado Data type: occurence This dataset is made available under the Open Database License (http://opendatacommons.org/licenses/odbl/1.0/). The Open Database License (ODbL) is a license agreement intended to allow users to freely share, modify, and use this Dataset while maintaining this same freedom for others, provided that the original source and author(s) are credited.

Supplementary material 2 Name and DNA sequences of pairs of primers used for amplification and sequencing of the CYTB gene Link: https://doi.org/10.3897/zookeys.697.15124.suppl2 Data type: molecular data This dataset is made available under the Open Database License (http://opendatacommons.org/licenses/odbl/1.0/). The Open Database License (ODbL) is a license agreement intended to allow users to freely share, modify, and use this Dataset while maintaining this same freedom for others, provided that the original source and author(s) are credited.Eliécer E. Gutiérrez, Kristofer M. Helgen, Molly M. McDonough, Franziska Bauer, Melissa T. R. Hawkins, Luis A. Escobedo-Morales, Bruce D. Patterson, Jesús E. Maldonado

Supplementary material 3 Supplementary information figure Link: https://doi.org/10.3897/zookeys.697.15124.suppl3 Authors: Eliécer E. Gutiérrez, Kristofer M. Helgen, Molly M. McDonough, Franziska Bauer, Melissa T. R. Hawkins, Luis A. Escobedo-Morales, Bruce D. Patterson, Jesús E. Maldonado Data type: statistical data This dataset is made available under the Open Database License (http://opendatacommons.org/licenses/odbl/1.0/). The Open Database License (ODbL) is a license agreement intended to allow users to freely share, modify, and use this Dataset while maintaining this same freedom for others, provided that the original source and author(s) are credited.
