# Supplementary material for: A gene-tree test of the traditional taxonomy of American deer: the importance of voucher specimens, geographic data, and dense sampling
Source: Zookeys. 2017 Sep 14;(697):87–131. doi: 10.3897/zookeys.697.15124 (PMC5673856; doi:10.3897/zookeys.697.15124)
Supplement: Supplementary material 1 — Gazetteer [file zookeys-697-087-s001.docx]

**Supplementary file 1. Gazetteer**. Below is a list of localities, species, and voucher specimens’ catalogue numbers corresponding to material used to generate sequences for the present study. All of the geographic information listed below was obtained directly from tags of museum specimens except when otherwise noted or when the text is placed within brackets. Localities are arranged alphabetically first by country, and then either by department (for Colombia, Honduras, Nicaragua), province (Ecuador), regions (Peru, Guyana), or state (Mexico, Venezuela, United States of America). Provenance localities are numbered to correspond to entries of Table 1 (in which GenBank numbers are also provided). Specimens indicated below are housed in the following institutions: American Museum of Natural History (AMNH; New York); Field Museum of Natural History (FMNH; Chicago); National Museum of Natural History (USNM; Washington DC); University of Kansas Natural History Museum (KU, Lawrence).

BRAZIL

(1) Mato Grosso: Descalvados [a label added, presumably by museum staff, also included “(= Descalvados Ranch)” as part of the locality], 142 m. *Ozotoceros bezoarticus* (FMNH 28297).

(2) Para: Bajao, Tocantins river. *Mazama nemorivaga* (AMNH 96171).

(3) São Paulo: Fazenda Varjão. *Blastocerus dichotomus* (FMNH 52329).

COLOMBIA

(4) Cauca: Guanacas, 3500 m. *Pudu mephistophiles* (AMNH 181505),

(5) Cundinamarca: Guasca, Río Balcones, 3000 m. *Mazama rufina* (FMNH 70563).

ECUADOR

(6) Los Ríos: Vinces. *Odocoileus virginianus* (AMNH 62872).

GUATEMALA

(7) Petén: 9 km NNW Chinajá. *Mazama temama* (KU 82215).

GUYANA

(8) Potaro-Siparuni: Iwokrama Reserve, 25 km WNW of Kurupukari, Burro Burro river. *Mazama gouazoubira* (KU 155307).

HONDURAS

(9) Cortes: La Lima. *Odocoileus virginianus* (KU 149129).

PERU

(10) Cajamarca: San Jacinto. *Mazama americana* (AMNH 67109).

(11) Puno: Rinconada, near Sandia. *Odocoileus virginianus* (FMNH 78421).

(12) Puno: Sandia, San Juan, 5000 ft [= 1524 m]. *Mazama chunyi* (FMNH 79912).

MEXICO

(13) Campeche: 42 km E Escárcega. *Odocoileus pandora* (KU 93857).

(14) Chihuahua: near Colonia García. *Odocoileus virginianus* (USNM 99351).

(15) Yucatán: 6 km N Tizimín. *Odocoileus virginianus* (KU 93852).

NICARAGUA

(16) Jinotega: San Rafael del Norte, Río Coco. *Odocoileus virginianus* (AMNH 29453).

UNITED STATES OF AMERICA

(17) Alaska: Kupreanof Id. *Odocoileus hemious* (USNM 249424).

(18) Arizona: Winslow (47 mi SW). *Odocoileus hemious* (USNM 99455).

(19) Washington DC: Smithsonian National Zoological Park, 3001 Connecticut Ave NW. *Odocoileus virginianus* [hybrid between *Od. virginianus* and *Od. hemionus*]. No voucher specimen but a tissue sample (WTD0028) available at the National Zoological Park, Smithsonian Institution.

VENEZUELA

(20) Bolívar: El Monaco, 59 km southeast of El Dorado. *Mazama nemorivaga* (USNM 374916).

(21) Yaracuy: San Felipe, 5 km N and 18 km W near Minas de Aroa. 500 m [note: locality information obtained from the museum’s online database; hence, it is likely that the information corresponds to the standardized locality as reported by the Smithsonian Venezuela Project (SVP). For specimens obtained by the SVP it is often possible to obtain more detailed locality data from specimen tags, but in the case of this particular specimen we were not able to access that information]. *Mazama americana* (USNM 443588).
