# Supplementary material for: A gene-tree test of the traditional taxonomy of American deer: the importance of voucher specimens, geographic data, and dense sampling
Source: Zookeys. 2017 Sep 14;(697):87–131. doi: 10.3897/zookeys.697.15124 (PMC5673856; doi:10.3897/zookeys.697.15124)
Supplement: Supplementary material 2 — Name and DNA sequences of pairs of primers used for amplification and sequencing of the CYTB gene [file zookeys-697-087-s002.docx]

**Supplementary file 2.** **Name and DNA sequences of pairs of primers used for amplification and sequencing of the CYTB gene.** We designed all primers listed below, with the following exceptions: LGL765 forward, LGL766 reverse, IDMAZ224L, and IDMAZH (see Gutiérrez et al. 2015 and references therein).

| **Primer name** | **Primer sequence** |
| --- | --- |
| **LGL765 forward** | 5´ GAAAAACCAYCGTTGTWATTCAACT |
| **CYTBPerR2** | 5´ TCAGCCGTAGTTGACGTCTC |
| **IDMAZ224L** | 5´ CATCCGACACAATAACAGCA |
| **IDMAZH** | 5´ TCCTACGAATGCTGTGGCTA |
| **GrayCYTB369L** | 5´ TTGGAGTAATTCTCCTATTCACAGT |
| **GrayCYTB478H** | 5´ AATCGGGTTAGGGTTGCTTT |
| **GrayCYTB503L** | 5´ TAGTTGAATGAATCTGAGGAGGCT |
| **GrayCYTB649H** | 5´ GTAGGGGTGGAATGGGATTT |
| **GrayCYTB613L** | 5´ CTTTTTCTCCACGAAACAGGA |
| **GrayRedCYTB764H** | 5´ GTGGGTTWGCTGGGGTGTAG |
|  |  |
| **GrayCYTB756L** | 5´ GCACCAGACCTACTCGGAGA |
| **RedCYTB898H** | 5´ ATGAAATAGGGGTATAAGGATTAGAA |
|  |  |
| **GrayCYTB756L** | 5´ GCACCAGACCTACTCGGAGA |
| **GrayCYTB901H** | 5´ TGTGGAGTAGAGGTATGAGAATCA |
|  |  |
| **GrayCYTB867L** | 5´ CGATCAATCCCAAATAAACTAGG |
| **GrayRedCYTB992H** | 5´ TCCGATTCATGTRAGTGTTAGTARG |
| **GrayRedCYTB981L** | 5´ CAGCCAATGTCTMTTCTGARY |
| **GrayCYTB1114H** | 5´ TTCATTTTAGGAGGTTGTTTTCGA |
|  |  |
| **GrayRedCYTB981L** | 5´ CAGCCAATGTCTMTTCTGARY |
| **GrayCYTB1114H** | 5´ TTCATTTTAGGAGGTTGTTTTCGA |
| **GrayRedCYTB981L** | 5´ CAGCCAATGTCTMTTCTGARY |
| **LGL766 reverse** | 5´ GTTTAATTAGAATYTYAGCTTTGGG |
